# Error mitigation enables PET radiomic cancer characterization on quantum computers

**DOI:** 10.1007/s00259-023-06362-6

**Published:** 2023-08-04

**Authors:** S. Moradi, Clemens Spielvogel, Denis Krajnc, C. Brandner, S. Hillmich, R. Wille, T. Traub-Weidinger, X. Li, M. Hacker, W. Drexler, L. Papp

**Affiliations:** 1 Applied Quantum Computing Group, Center for Medical Physics and Biomedical Engineering, Medical University of Vienna, Waehringer Guertel 18-20, T1090 Vienna, Austria; 2 Division of Nuclear Medicine, Medical University of Vienna, Vienna, Austria; 3 Center for Medical Physics and Biomedical Engineering, Medical University of Vienna, Vienna, Austria; 4 Institute for Integrated Circuits, Johannes Kepler University Linz, Linz, Austria; 5 Chair for Design Automation, Technical University of Munich, Munich, Germany

**Keywords:** Cancer, Quantum computing, Machine learning, Radiomics, PET

## Abstract

**Background:**

Cancer is a leading cause of death worldwide. While routine diagnosis of cancer is performed mainly with biopsy sampling, it is suboptimal to accurately characterize tumor heterogeneity. Positron emission tomography (PET)-driven radiomic research has demonstrated promising results when predicting clinical endpoints. This study aimed to investigate the added value of quantum machine learning both in simulator and in real quantum computers utilizing error mitigation techniques to predict clinical endpoints in various PET cancer patients.

**Methods:**

Previously published PET radiomics datasets including 11C-MET PET glioma, 68GA-PSMA-11 PET prostate and lung 18F-FDG PET with 3-year survival, low-vs-high Gleason risk and 2-year survival as clinical endpoints respectively were utilized in this study. Redundancy reduction with 0.7, 0.8, and 0.9 Spearman rank thresholds (SRT), followed by selecting 8 and 16 features from all cohorts, was performed, resulting in 18 dataset variants. Quantum advantage was estimated by Geometric Difference (GD_Q_) score in each dataset variant. Five classic machine learning (CML) and their quantum versions (QML) were trained and tested in simulator environments across the dataset variants. Quantum circuit optimization and error mitigation were performed, followed by training and testing selected QML methods on the 21-qubit IonQ Aria quantum computer. Predictive performances were estimated by test balanced accuracy (BACC) values.

**Results:**

On average, QML outperformed CML in simulator environments with 16-features (BACC 70% and 69%, respectively), while with 8-features, CML outperformed QML with + 1%. The highest average QML advantage was + 4%. The GD_Q_ scores were ≤ 1.0 in all the 8-feature cases, while they were > 1.0 when QML outperformed CML in 9 out of 11 cases. The test BACC of selected QML methods and datasets in the IonQ device without error mitigation (EM) were 69.94% BACC, while EM increased test BACC to 75.66% (76.77% in noiseless simulators).

**Conclusions:**

We demonstrated that with error mitigation, quantum advantage can be achieved in real existing quantum computers when predicting clinical endpoints in clinically relevant PET cancer cohorts. Quantum advantage can already be achieved in simulator environments in these cohorts when relying on QML.

**Supplementary Information:**

The online version contains supplementary material available at 10.1007/s00259-023-06362-6.

## Introduction

To date, cancer remains one of the major causes of deaths worldwide, as in 2020, approximately 20 million new cases were identified. The routine diagnosis of cancer is performed by invasive biopsy sampling which is considered inaccurate [1], given that cancers are heterogeneous; thus, small biopsy samples cannot accurately characterize the whole stage of the given lesion [2]. In addition, biopsies are painful, increase risk of infection, and in general, may reduce the quality of life of patients [3]. Positron emission tomography (PET)/computer tomography (CT) and recently PET/magnetic resonance imaging (MRI) hybrid imaging techniques have been playing a crucial role in in vivo cancer detection and characterization settings [4–6]. Radiomics is the process of extracting numerical features from medical images in order to characterize diseases in vivo [7]. Recent advancements in the field of PET radiomics combined with machine learning approaches have remonstrated promising results in predicting clinical end-points [2, 4, 8–10]. Nevertheless, radiomic models are challenged by factors related to metabolic variations across patients in PET, as well as variations in imaging, delineation, and radiomic feature extraction parameters [7]. While the Imaging Biomarker Standardization Initiative (IBSI) [11] has helped to standardize the process of performing feature extraction from medical images, the recently reported joint EANM/SNMMI guideline for radiomics in nuclear medicine lays out the foundations of quantitative radiomics on a wide spectrum of analysis aspects to characterize diseases in vivo [7]. Still, various challenges remain on the level of small training datasets, combined with complex and difficult-to interpret prediction models that do not support the process of clinical adoption. Consistently, to date, the wide-scale clinical adoption of AI-driven approaches relying on PET in cancer patients is yet to be witnessed [12].

Quantum computing is an emerging field with the promise to revolutionize computationally complex problems such as modeling and simulation, optimization, and artificial intelligence (AI) [13]. While classical computers operate with bits that can either have values 0 or 1, quantum computing operates with qubits that can represent both 0 and 1 values with a probabilistic outcome, by encoding complex information [13, 14]. Relying on quantum phenomena such as interference, superposition, and entanglement, the so-called quantum circuits can model complex real-life computational problems with simple qubit gate calculations [15]. While the public perception of quantum advantage is associated to superior computing speed, quantum advantage has many different forms. Specifically, one may encode an N-dimensional vector to log_2_*N* number of qubits, which results in speedup as well as in a much simpler quantum algorithmic complexity compared to its classic computing counterpart [13, 16]. This simplified search space naturally aids the training process of quantum machine learning (QML) approaches compared to their classic computing counterparts [13, 14]. Consistently, various QML studies have demonstrated the feasibility to both estimate [14] and to achieve [13, 17, 18] a higher predictive performance when relying on quantum ML approaches compared to classic ML [19]. Recently, it has also been demonstrated that QML requires less training data than classic ML does to build high-performing predictive models [20]. To date, various quantum algorithms (a.k.a. quantum circuits) have been proposed on existing,﻿ so-called noisy intermediate scale quantum computers (NISQ) [15]. Nevertheless, most problem fields cannot efficiently utilize NISQs due to their low qubit count and high noise levels [15]. Ongoing activities in this regard focus on proposing and implementing error mitigation techniques that can counter-balance quantum gate as well as measurement errors [21–24]. In general, the majority of quantum computing research focuses on extending the number of qubits and minimizing noise in future quantum hardware and tend to underestimate the importance of existing NISQs as they are challenging to scale [15]. In contrast, the mentioned advantageous properties of quantum computing render it an interesting candidate to further advance PET radiomic research.

In light of the challenges radiomics and machine learning is facing in the field of cancer research, we hypothesize that by relying on existing NISQs combined with novel error mitigation techniques, quantum advantage can be achieved in clinically relevant cancer cohorts. Therefore, this study had the following objectives: (a) to compare classic and quantum ML predictive performances relying on cross-validation techniques when predicting clinical endpoints in various cancer patients; (b) to investigate whether the magnitude of quantum advantage in light of QML predictive performance can be accurately estimated in the cancer datasets prior to engaging with NISQs; and (c) to investigate the feasibility of utilizing a real quantum computer combined with novel error mitigation techniques for QML prediction in the collected cancer datasets.

## Methods

### Dataset

**Fig. 1:**
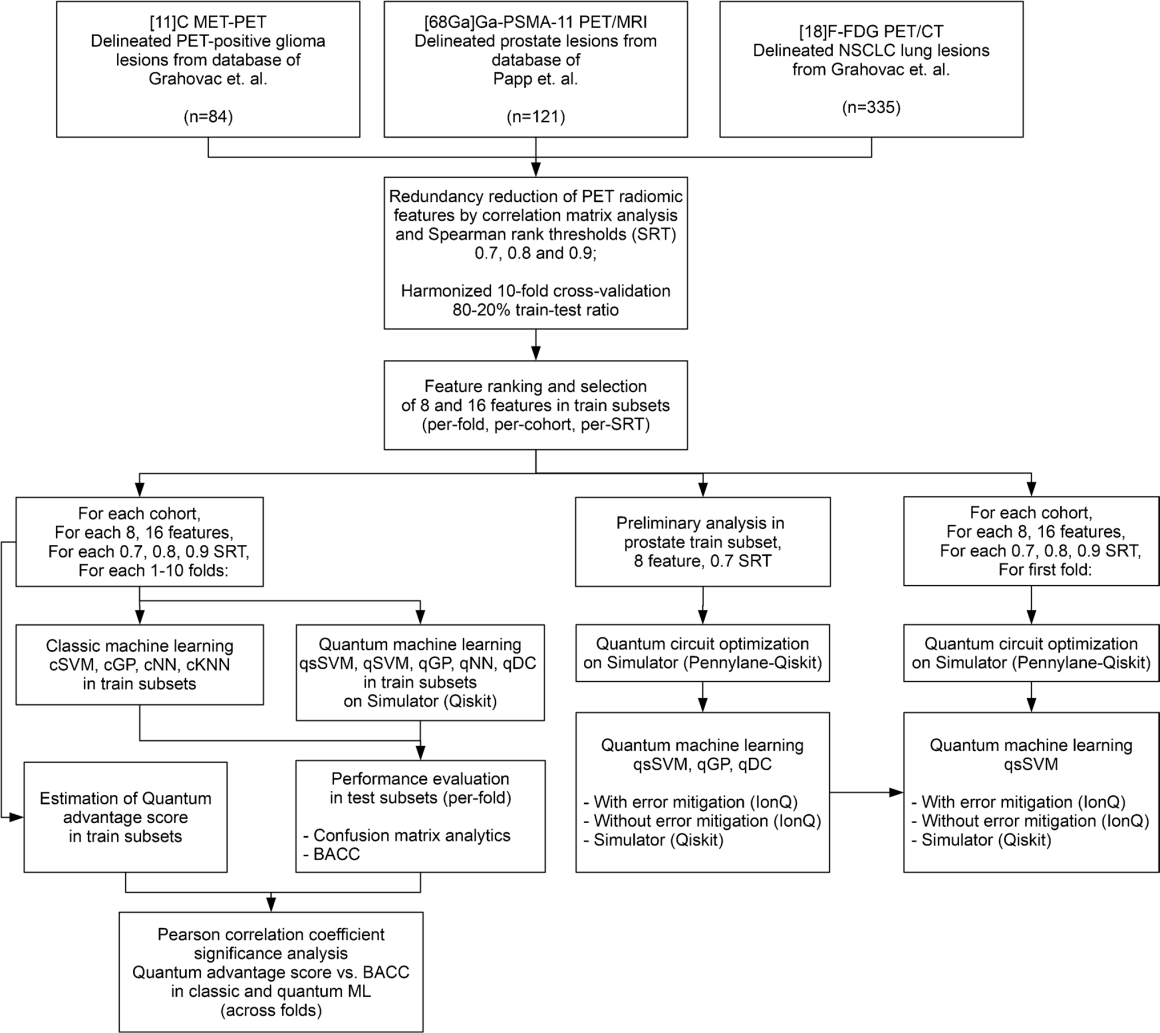
CONSORT diagram of the study. Overall, three retrospectively available and previously reported cohorts were included, composed of Imaging Biomarker Standardization Initiative (IBSI) [11] conform PET radiomic features to predict cohort-specific clinical endpoints, specifically, Gleason low-vs-high risk from prostate, 3-year survival from glioma and 2-year survival from lung cancer cases. After performing redundancy reduction via correlation matrix and Spearman ranking thresholds (SRT) of 0.7, 0.8, and 0.9 [9], each dataset with its SRT-specific variant undergoes a tenfold cross-validation train-test split with 80–20% train-test ratio. The cross-validation split is harmonized, to maintain the same split across the different SRT variants. Feature selection with R-squared ranking is performed in the train subsets to result in eight and 16 features per-cohort, per-SRT and per-fold [28]. Estimation of quantum advantage scores across the train subsets is performed via Geometric Difference (GD) score [14]. In addition, classic (CML) and quantum machine learning (QML) prediction models are built to predict clinical endpoints based on the training subsets of data variants. Predictive performance comparison of CML and QML methods is performed through the test subsets of the cross-validation scheme relying on confusion matrix analytics and balanced accuracy (BACC). Statistical significance in-between the GD scores and the test BACC values of QML approaches across the tenfolds is performed by Pearson correlation coefficient analysis. Preliminary analysis is performed with the first fold of the prostate cohort with 8-features and 0.7 SRT where quantum circuit optimization is done in simulator environment, followed by building prediction models of selected QML methods in the 21-qubit IonQ Aria real quantum device with and without error mitigation techniques. Predictive performance estimation with and without error mitigation as well as in a noiseless simulator environment is performed on the test subsets. The best performing QML approach with error mitigation is selected and the first fold of all cohorts and their 8, 16 feature as well as their SRT variants undergo the same 21-qubit IonQ Aria evaluation with and without error mitigation. cSVM — classic kernel support vector machine; cGP — classic kernel Gaussian process; cNN — classic neural network; cKNN — classic *k*-nearest neighbor; qsSVM — simplified quantum kernel support vector machine; qSVM — quantum kernel support vector machine; qGP — quantum kernel Gaussian process; qNN — quantum neural network; qDC — quantum distance classifier

This study relied on a three, previously published PET radiomic cancer cohorts. All cohorts were composed of imaging biomarker standardization initiative (IBSI)-conform PET radiomic features [11] and their respective clinical endpoints to predict: A [^68^ Ga]Ga-PSMA-11 (PSMA-11) radiomic dataset containing 121 delineated lesions with low-vs-high lesion risk [2, 9], 84 11C-Methionine (MET) glioma cases with 3-year survival [9, 25], and 335 18F-FDG PET lung cases with 2-year survival clinical endpoints [9, 26]. All radiomic datasets underwent redundancy reduction with correlation matrix analysis and Spearman rank thresholds (SRT) of 0.7, 0.8, and 0.9 [9], where in case of redundant Spearman rank clusters, the feature with the highest variance was selected. For detailed patient characteristics and the IBSI-conform imaging and radiomic steps, see the respective reports of the cohorts. For the CONSORT diagram of this study, see Fig. 1. For the Checklist for Artificial Intelligence in Medical Imaging (CLAIM) guidelines [27], see Supplemental K.

### Cross-validation scheme

Tenfold cross-validation scheme with 80:20% train-to-test ratio in each cohort was utilized to estimate predictive model performances in this study. The train-test split was performed to ensure that no sample from the same patient is assigned to the given fold’s train and test split concurrently in order to avoid patient-level data leakage [9]. The test set was balanced in each fold for all cohorts.

### Feature ranking and selection

Feature ranking and selection of 8 as well as 16 features in each training subset of each cohort and their three SRT variants was performed for further analysis [28]. These numbers were chosen to satisfy two requirements: on the one hand, to minimize the chances of overfitting while building predictive models according to the curse of dimensionality rule [10, 29]. On the other hand, to ensure that the number of features encoded to quantum circuits is 2^*N*^ (*N* > 1), which was to result in an optimal number of qubits for the chosen classic-to-quantum encoding step and subsequent quantum machine learning (QML) [30]. In case the given SRT resulted in less than 8 or 16 number of features in any of the cohorts, zero-padding was applied before encoding to the required number of quantum bits [31]. After performing the feature ranking and selection phase, this study had 3 cohorts × 3 SRT × 2 feature/qubit count = 18 dataset variants.

### Quantum machine learning

Quantum machine learning requires classical data to be encoded to quantum states. In order to minimize quantum circuit complexity (a.k.a. the number of quantum gate operations), the encoding circuit itself can be optimized. For quantum encoding optimization, this study relied on the Limited memory Broyden–Fletcher–Goldfarb–Shanno (LBFGS) method executed on the Qiskit-Pennylane simulator in combination with PyTorch-compatible quantum nodes (see Appendix B of the supplementary material for details of our tested encoding optimization strategies). The amplitude encoding scheme was utilized to encode 8 features to log_2_(8) = 3 and 16 features to log_2_(16) = 4 number of qubits, respectively [30, 32].

After encoding, the quantum state processing circuit parts were optimized by gate decomposition approaches to minimize the number of gates manifesting superposition, entanglement, and interference for QML (see Appendix C of the supplement for details). QML approaches were utilized to train quantum predictive models in each fold relying on the Qiskit-Azure simulator environment considering IonQ simulator as backend [33]: A simplified quantum kernel support vector machine (qsSVM) [13], a quantum kernel support vector machine (qSVM) utilizing optimization [34, 35], a quantum kernel Gaussian process (qGP) [36], a quantum neural network (qNN) [37], and a quantum distance classifier (qDC) [13]. The two quantum SVM variants were involved to investigate the effect of optimization vs. no optimization in-between qSVM and qsSVM approaches, respectively (see Appendix D and E for details of the utilized QML approaches including their quantum circuit diagrams, behavior, and parameter sets and see Sec. Access for accessing the source code utilized to build QML prediction models for this study). For detailed parameters of quantum ML algorithms, see Appendix F of the supplement.

### Classic machine learning

Classic kernel support vector machine (cSVM) [38], classic kernel Gaussian process (cGP) [39], classic neural network (cNN) [40], and classic *k*-nearest neighbor (ckNN) [41] machine learning approaches were built on the training subsets across the cross-validation scheme relying on the Python software package scikit-learn [42]. The choice of these approaches was followed by guidelines as reported in [43] in order to ensure a fair comparison of quantum-classic ML approaches. The ckNN approach was the classic variant of qDS with the difference of qDS operating with *k* = 1 and ckNN with *k* = 5 nearest neighbors. For detailed parameters of classic ML algorithms, see Appendix F of the supplementary material (see Sec. Access for accessing the source code utilized to build CML prediction models for this study).

### Performance evaluation

All quantum and classic ML models built in this study were cross-validated by relying on the same cross-validation split configurations of all 18 dataset variants to avoid test performance fluctuations due to method-specific random splits. Each quantum and classic predictive models were evaluated based on the given test set samples by the calculation of confusion matrix (CM) analytics per-model and per-fold in the given dataset variant. The number of true positive (TP), false positive (FP), true negative (TN), and false negative (FN) cases was calculated for each confusion matrix, from which balanced accuracy (BACC) was calculated. The mean BACC were calculated together with their 95% confidence intervals (CI) across the tenfolds for each quantum and classic predictive models.

### Estimation of quantum advantage

The Geometric Difference (GD_Q_) score was utilized to estimate the magnitude of quantum advantage [14] in each tenfold training subsets of the 18 dataset variants, relying on the Qiskit simulator environment [44]. The GD_Q_ is a measurement which characterizes the power of data regarding quantum ML predictability without the need to engage with any actual quantum computers and QML approaches. For estimation of the Geometric Difference scores and its physical meaning, see Appendix A of the supplement. In case GD_Q_ > 1.0, a higher predictive performance is likely in quantum ML models compared to their CML counterparts. In case GD_Q_ ≤ 1.0, the given dataset will not result in higher QML predictive performance compared to CML [14]. The average QML and CML test balanced accuracy values (qBACC and cBACC, respectively) were calculated across test subsets of the cross-validation folds and the difference balanced accuracy (dBACC = qBACC – cBACC) was calculated. The significance of dependency in-between GD_Q_ and dBACC was calculated by Pearson correlation coefficient analysis where *p* < 0.05 was considered as significance threshold. In addition to the above, we estimated the required amount of classical computing needs to approximate GD_Q_ = 1.0 compared to surpassing it with quantum ML (see Appendix A of the supplement for details).

### Quantum error mitigation

Since existing NISQ devices are noisy, their outputs are subjects of both gate and measurement errors [15, 45]. Therefore, the output needs to undergo error mitigation. This process can be described as a classical regression problem between noiseless simulator vs. noisy NISQ measurement values. This study utilized a classical random forest regression algorithm [42] to mitigate quantum errors on the 21-qubit IonQ Aria device (see Appendix G of the supplement for more details about the IonQ quantum device). In order to estimate the effect of the proposed error mitigation technique and to identify which QML approach benefits the most from such technique, a two-step approach was followed. First as a preliminary analysis, the first fold from the prostate cohort with 8 features and 0.7 SRT was selected and the 21-qubit IonQ Aria quantum computer was utilized to train and test qDC, qGP, and qsSVM prediction models with and without error mitigation (EM). The qSVM and qNN approaches were excluded from this step due to resource constraints when relying on the IonQ device. For details of the EM techniques, see Appendix H. The test balanced accuracy (BACC) performance evaluation results of qDC, qGP, and qsSVM relying on the simulator environment were compared to the 21-qubit IonQ device test BACC results to estimate the effect of EM. The second step selected the QML approach which yielded the highest test BACC on the IonQ device considering both no EM and with EM, and repeated the analysis on the first fold of all the 18 dataset variants on the IonQ device with and without EM.

## Results

### Performance evaluation

Predictive performance evaluation over test subsets of the utilized cross-validation scheme revealed that on average, both QML and CML approaches yielded 64–73% balanced accuracy (BACC) ranges across all cohorts and their SRT configurations with 8-features and 3-qubits. Nevertheless, CML outperformed QML in 5 out of 9 experiments, QML outperformed CML in 1 case and in 3 cases, the BACC was equal in-between QML and CML (Table 1). In contrast, the QML and CML approaches in case of 16-features and 4-qubits yielded an average BACC range of 64–78% and 64–75%, respectively. Here, QML outperformed CML in 5 out of 9 cases, while CML outperformed QML in 2 cases and in 2 cases QML and CML yielded identical BACC results (Table 2). On average, confidence intervals (CI) across all QML and CML methods were 3.58 for QML and 3.91 for CML with 8-features and 3-qubits, and they were 2.86 for QML and 3.85 for CML with 16-features and 4-qubits. The highest average QML test BACC advantage was + 4% with 16-features and 4-qubits, respectively (see Tables 1 and 2 for detailed predictive performance evaluation results of the included quantum and classic ML approaches with 8 and 16 features (3 and 4 qubits, respectively)).

**Table 1:** Test balanced accuracy (BACC) predictive performance values of quantum and classic ML approaches in the collected cohorts and their three different Spearman rank threshold variants (0.7, 0.8, and 0.9) with 8 features (3-qubits) executed in noiseless simulator environments. Quantum ML and classic ML-specific values represent average (*µ*) test BACC and their respective 95% confidence intervals (CI) across the tenfolds of each respective dataset configuration. GD_Q_ represents the average Geometric Difference score across tenfolds of the given dataset variant. Average QML and average CML *µ* and CI values (bold) represent the average of all respective QML and CML values in the given dataset variant. *SRT*, Spearman rank threshold; *qDC*, quantum distance classifier; *qNN*, quantum neural network; *qsSVM*, simplified quantum kernel support vector machine; *qSVM*, quantum kernel support vector machine; *qGP*, quantum kernel Gaussian process; *cKNN*, classic *k*-nearest neighbor; *cNN*, classic neural network; *cSVM*, classic kernel support vector machine; *cGP*, classic kernel Gaussian process

8 features/3-qubits		Glioma			Lung			Prostate		
	SRT	0.9	0.8	0.7	0.9	0.8	0.7	0.9	0.8	0.7
	GD_Q_	3.75	0.66	0.55	5.63	0.52	0.74	0.87	0.48	0.46
qDC	*µ*	69	66	59	68	64	65	68	70	66
	CI	2.49	2.93	5.68	3.85	2.12	3.89	3.37	3.56	3.80
qNN	*µ*	73	73	63	73	67	63	65	64	63
	CI	5.99	4.25	3.61	3.32	3.97	2.93	1.97	2.47	2.08
qsSVM	*µ*	73	71	71	73	68	64	67	65	65
	CI	5.47	4.39	5.58	3.05	2.96	2.58	1.78	1.36	2.38
qSVM	*µ*	78	74	68	73	67	65	67	67	65
	CI	7.32	3.81	5.68	3.32	4.40	2.71	2.32	3.20	2.67
qGP	*µ*	73	72	62	68	65	64	69	67	66
	CI	4.07	5.55	4.99	4.21	4.32	2.60	2.47	2.32	3.32
Average QML	*µ*	**73**	**71**	**65**	**71**	**66**	**64**	**67**	**67**	**65**
	CI	**5.07**	**4.19**	**5.11**	**3.55**	**3.55**	**2.94**	**2.38**	**2.58**	**2.85**
ckNN	*µ*	67	70	66	67	69	63	69	70	65
	CI	5.54	4.36	7.08	3.57	3.33	2.18	2.80	3.27	3.03
cNN	*µ*	71	75	71	75	70	65	65	66	67
	CI	5.02	4.22	6.56	3.10	3.24	5.16	1.97	3.04	3.94
cSVM	*µ*	72	75	78	73	70	65	67	67	67
	CI	5.55	4.22	4.99	2.89	3.56	2.46	1.78	4.21	3.27
cGP	*µ*	73	73	68	68	68	65	71	67	67
	CI	5.86	4.90	5.14	4.11	4.11	3.59	1.97	2.59	4.16
Average CML	*µ*	**71**	**73**	**71**	**71**	**69**	**64**	**68**	**67**	**67**
	CI	**5.49**	**4.42**	**5.94**	**3.42**	**3.56**	**3.35**	**2.13**	**3.28**	**3.60**

**Table 2:** Test balanced accuracy (BACC) predictive performance values of quantum and classic ML approaches in the collected cohorts and their three different Spearman rank threshold variants (0.7, 0.8, and 0.9) with 16 features (4-qubits) executed in noiseless simulator environments. Quantum ML and classic ML-specific values represent average (*µ*) test BACC and their respective 95% confidence intervals (CI) across the tenfolds of each respective dataset configuration. GD_Q_ represents the average Geometric Difference score across tenfolds of the given dataset variant. Average QML and average CML *µ* and CI values (bold) represent the average of all respective QML and CML values in the given dataset variant. *SRT*, Spearman rank threshold; *qDC*, quantum distance classifier; *qNN*, quantum neural network; *qsSVM*, simplified quantum kernel support vector machine; *qSVM*, quantum kernel support vector machine; *qGP*, quantum kernel Gaussian process; *cKNN*, classic *k*-nearest neighbor; *cNN*, classic neural network; *cSVM*, classic kernel support vector machine; *cGP*, classic kernel Gaussian process

16 features/4-qubits		Glioma			Lung			Prostate		
	SRT	0.9	0.8	0.7	0.9	0.8	0.7	0.9	0.8	0.7
	GD_Q_	3.20	2.43	2.33	5.21	2.47	1.08	8.33	7.17	1.36
qDC	*µ*	74	70	69	70	68	64	70	68	69
	CI	2.93	0.04	4.25	2.56	2.71	1.51	2.67	4.29	3.62
qNN	*µ*	76	73	73	71	70	63	68	65	66
	CI	3.81	0.02	3.27	2.06	3.81	3.18	2.08	3.77	2.72
qsSVM	*µ*	79	73	69	70	69	64	65	65	66
	CI	3.65	0.05	5.47	2.19	2.32	2.58	1.19	2.32	3.03
qSVM	*µ*	78	77	77	74	70	63	68	65	68
	CI	3.61	0.03	4.07	2.92	4.17	2.98	2.30	2.98	3.37
qGP	*µ*	83	74	66	73	72	66	70	67	65
	CI	4.52	0.04	5.14	3.56	3.69	2.88	4.25	2.91	3.03
Average QML	*µ*	**78**	**73**	**71**	**72**	**70**	**64**	**68**	**66**	**67**
	CI	**3.71**	**0.04**	**4.44**	**2.66**	**3.34**	**2.62**	**2.50**	**3.25**	**3.16**
ckNN	*µ*	74	66	62	70	67	63	69	66	67
	CI	5.68	5.68	6.06	3.65	4.44	2.70	2.47	3.03	3.94
cNN	*µ*	73	71	75	76	70	64	68	65	68
	CI	4.90	4.39	4.87	3.60	3.56	4.47	4.20	3.71	3.37
cSVM	*µ*	74	75	74	75	70	64	68	65	65
	CI	5.68	0.05	6.18	3.72	3.98	4.57	3.98	3.53	2.72
cGP	*µ*	80	71	64	74	70	65	69	61	66
	CI	4.36	0.05	4.90	3.37	3.76	2.70	4.16	2.74	3.32
Average CML	*µ*	**75**	**71**	**69**	**73**	**69**	**64**	**69**	**64**	**67**
	CI	**5.16**	**2.54**	**5.50**	**3.59**	**3.93**	**3.61**	**3.70**	**3.25**	**3.34**

### Estimation of quantum advantage

**Fig. 2:**
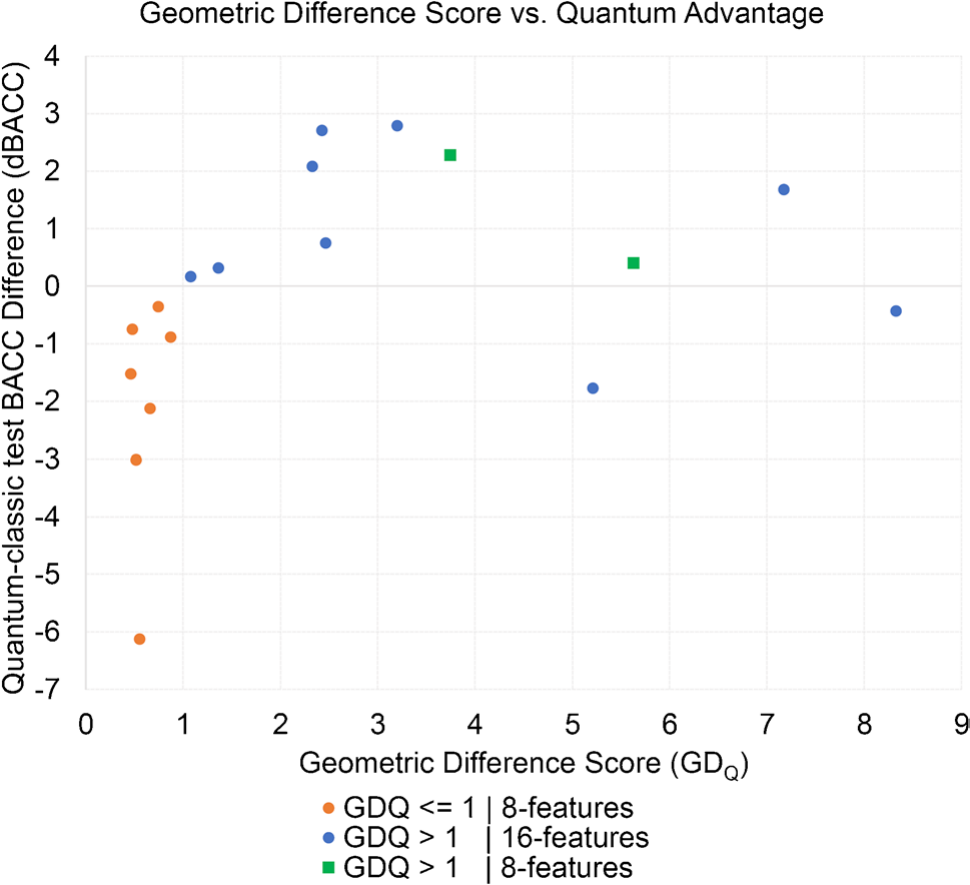
Average Geometric Difference (GD_Q_) scores (across folds per-cohort and per-SRT) and average quantum-classic balanced accuracy differences (dBACC) plotted across the collected cohorts and their configurations (8 and 16 features, 0.7, 0.8, and 0.9 SRT). Results confirm that in case GD_Q_ ≤ 1.0, quantum advantage is not achieved in 7 out of 7 experiments, and that all those cases are with 8-features (orange), while in case of GD_Q_ > 1.0, quantum advantage can be achieved 9 out of 11 experiments. The majority of these cases are with 16-features (blue), while two cases are with 8-features (green)

The tenfold averaged GD_Q_ scores across all cohorts and their three SRT variants was 0.48–5.63) with 8-features and 3-qubits (Table 1). In contrast, the tenfold averaged GD_Q_ scores within 16-features and 4-qubits were 1.08–7.17 (Table 2). These GD_Q_ distributions were in line with the respective QML-vs-CML predictive performances across the 8-feature, 3-qubit and the 16-feature, 4-qubit experiments (see Section “Performance evaluation”). Results of the Pearson correlation indicated that there is a non-significant medium positive relationship between GD_Q_ and dBACC (*r* = 0.366, *p* = 0.136). When differentiating cases with the GD_Q_ ≤ 1.0 threshold, the relationship of the GD_Q_ scores and dBACCs across the experiments become more prominent. As such, all GD_Q_ ≤ 1.0 cases were all with 8-features and 3-qubits, and in these cases, QML did not yield superior performance over CML (Fig. 2). In case GD_Q_ > 1.0, QML on average outperformed CML in 9 out of 11 dataset variant executions. The two 16-feature 4-qubit experiments that failed in light of GD_Q_ > 1.0 and QML advantage were associated to 0.9 SRT from the lung and prostate datasets, respectively. According to our classical kernel-based ML algorithms, the prediction performance which could become comparable to QML in light of GD_Q_ requires classical kernel calculations with approximately 10-times higher complexity compared to QML kernels (see Appendix A of the supplement).

### Quantum error mitigation

**Fig. 3:**
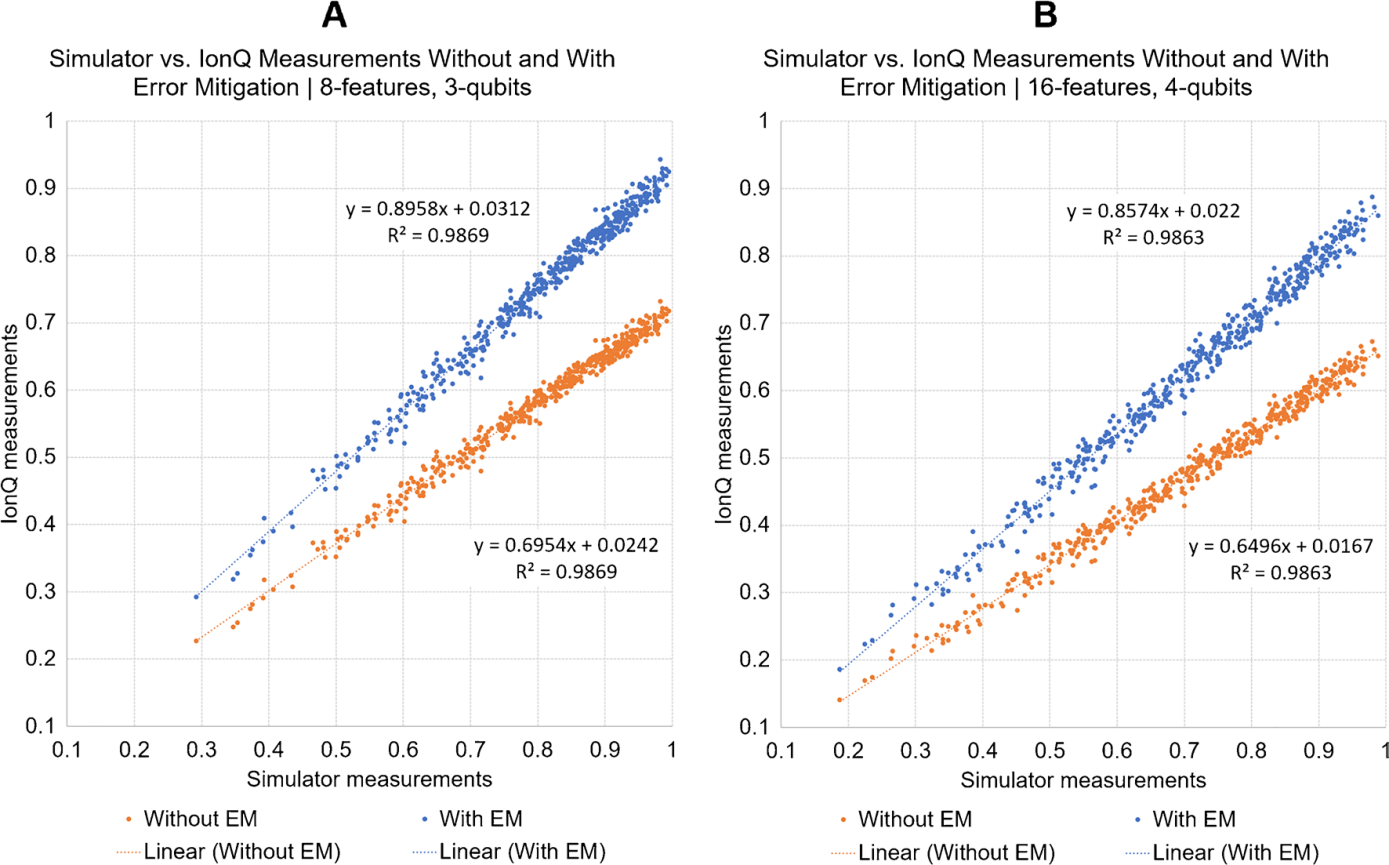
Examples of noiseless simulator vs. IonQ device measurements without and with error mitigation (EM) in the first fold of the prostate dataset with 8 (**A**) and 16 (**B**) features (3 and 4 qubits, respectively), and with 0.7 Spearman rank threshold. Note that the *x*-axis is aligned to the range of the simulator measurements, while the *y*-axes are specific to the given IonQ measurements. Units of both *x-* and *y*-axes denote the inner product of train and test sets after encoding. These values move in the range of 0.0–1.0. Value 0.0 means that there is no correlation between the given train and test sample, while value 1.0 means that they perfectly correlate. The distribution of the plots implies how similar simulator and measured results are. A perfect noiseless distribution would map the coordinates to the diagonal (*y* = *x*) of the plot

The average test balanced accuracy (BACC) in noiseless simulator environments was 72.73% for qsSVM, qGP, and qDC approaches in the selected prostate train-test split. In contrast, the test BACC on the IonQ device without error mitigation (EM) was in the range of 59.09–68.18% for the selected QML methods. Utilizing the proposed EM technique on IonQ yielded identical results to the noiseless simulator test performances in the qsSVM and qDC algorithms (Table 3). Since qsSVM had the highest BACC when considering both no EM and with EM cases, this method was utilized in the second evaluation phase, where all 18 dataset variants were involved in the analysis on IonQ with and without EM. Here, all 18 except two error mitigated qsSVM test BACC results were identical with the noiseless simulator environments. The two cases where predictive performance decreased were in the prostate cohort with 0.8 SRT in both 8 and 16 feature variants. On average, IonQ test BACC performance values without EM were 69.94%, while they were 75.66% with EM (76.17 in noiseless simulator environments) (see Table 4 for the detailed comparative results and see Fig. 3 for an example inner product plot with 8 and 16 features (3 and 4 qubits, respectively) without and with EM vs. noiseless simulator environments).

**Table 3:** Test balanced accuracy (BACC) values of qsSVM, qGP, and qDC quantum ML approaches in noiseless simulator environments and on the IonQ Aria device without error mitigation (EM) and with EM. The dataset used for the analysis was the first fold from the prostate cohort, 8 features (3 qubits) and Spearman rank threshold 0.7. *qsSVM*, simplified quantum kernel support vector machine; *qGP*, quantum kernel Gaussian process; *qDC*, quantum distance classifier

QML approach	Qiskit simulator	IonQ no EM	IonQ with EM
qsSVM	72.73	68.18	72.73
qGP	72.73	59.09	68.18
qDC	72.73	63.64	72.73

**Table 4:** Test qsSVM balanced accuracy (BACC) performance values of all included cohorts, feature counts, and their Spearman rank threshold variants when relying on noiseless simulator, the IonQ Aria device without error mitigation (EM) and with EM. BACC values are measured from the first fold of each dataset variant in a hold-out train-test scenario. Last row (bold) demonstrates the average BACC across all cohorts and feature/qubit configurations in the given quantum environment

Feature/qubit count	Cohort	SRT	Simulator	No EM	With EM
16 features/4 qubits	Glioma	0.9	83.33	75.00	83.33
		0.8	83.33	75.00	83.33
		0.7	83.33	66.67	83.33
	Lung	0.9	73.08	73.08	73.08
		0.8	73.08	73.08	73.08
		0.7	71.15	69.23	71.15
	Prostate	0.9	72.73	68.18	72.73
		0.8	72.73	63.64	68.18
		0.7	72.73	68.18	72.73
8 features/3 qubits	Glioma	0.9	83.33	75.00	83.33
		0.8	83.33	75.00	83.33
		0.7	83.33	75.00	83.33
	Lung	0.9	76.92	73.08	76.92
		0.8	76.92	71.15	76.92
		0.7	63.46	57.69	63.46
	Prostate	0.9	72.73	68.18	72.73
		0.8	72.73	63.64	68.18
		0.7	72.73	68.18	72.73
Average			**76.17**	**69.94**	**75.66**

## Discussion

In this study, we proposed a comprehensive approach to optimize quantum circuits combined with error mitigation. These approaches made quantum machine learning (QML) in clinically relevant PET radiomic datasets feasible in both simulator and real quantum hardware. In addition, we compared results derived using QML with their classic ML (CML) counterparts, while ensuring a fair comparison by following the guidelines in [43].

Our findings confirm that quantum advantage can be efficiently estimated without engaging with quantum computing by relying on the previously proposed geometric difference (GD_Q_) score as defined in [14]. According to the above, we found that in case GD_Q_ > 1.0, QML can outperform CML already in simulator environments with up to + 4% balanced accuracy (BACC) and with a narrowed confidence interval (CI), implying improved robustness of QML. Furthermore, our quantum circuit optimization and error mitigation approaches resulted in feasible QML circuit evaluations in real quantum hardware when relying on simple circuits and minimum amount of circuit measurements.

On average, the Geometric Difference (GD_Q_) scores through the dataset variants were higher than 1.0 with 16-features and 4-qubits, implying a high likelihood of achieving QML advantage. When GD_Q_ > 1.0 and QML failed to overperform CML, the underlying dataset had SRT 0.9, which is logical, as such a high SRT increases the number of redundant radiomic features, thus, the chances of overfit [7, 46, 47]. At the same time, all inferior QML approaches that had GD_Q_ ≤ 1.0 were built with 8-features and 3-qubits. Correlating GD_Q_ scores with QML-CML relative test BACCs demonstrated that in case GD_Q_ > 1.0, relatively high GD_Q_ scores (e.g., > 5.0) do not necessarily yield higher-performing QML approaches compared to CML. According to the above, proposing feature ranking approaches for QML solely building on the maximization of GD_Q_ is not advised, as feature redundancy and ML-specific behavior also have to be accounted for. Our study also found that with the utilized kernels, in case classical GD_Q_ < 1.0, CML can only achieve the same result as QML with a GD_Q_ ~ 1.0 on the expense of approximately 10-times more computational costs. In general, quantum encoding does not create additional or added information from classical data, but the encoding step itself may transform classical data into quantum states where the data is better separable. This phenomena can be estimated by the GD_Q_ score. Overall, we wish to emphasize that GD_Q_ is the property of the data and not the QML or CML algorithm; hence, a weak correlation of increasing GD_Q_ vs. QML BACC (*p* = 0.136) was demonstrated in our experiments.

When comparing the overall test performance of QML and CML methods relying on the cross-validation scheme, we identified a clear trend towards a higher BACC and in comparison with CML while increasing robustness in CI ranges as well.

Test predictive performance comparison of QML algorithms revealed that quantum kernel methods (3-qubits BACC: 62–78%, 4-qubits BACC: 63–83%) were outperforming qNN (3-qubits BACC: 63–73%, 4-qubits BACC: 63–76%), which is in accordance to prior findings, demonstrating that quantum kernel-based training models can solve supervised classification tasks better or equally than qNN learning models for small data samples [17]. The above measurements were performed in simulator environments that are executed on classical hardware and software. This implies that quantum advantage in clinically relevant radiomic datasets may be achieved without the need to use real quantum hardware. Nevertheless, this is only true due to the low feature and qubit counts as well as the relatively small data size which is a generic property of many cancer cohorts [7]. Indeed, this property of QML advantage has been demonstrated in other studies [20]. A low feature count also supports the explainability of ML models and the process of biomarker identification in general [7, 47].

While the above quantum advantage in simulator environments is encouraging, it is important to understand that real, noiseless quantum hardware may provide a higher fidelity than any simulator. Over time, NISQs will become less and less noisy. Real quantum hardware has properties such as interference, superposition, and entanglement that cannot be simulated on a classical hardware with the same fidelity. This implies that future noiseless or error-corrected quantum hardware has the potential to further advance the predictive performance of QML approaches, when exploiting quantum phenomena. This, however, will have to be evaluated and confirmed as part of future research, once noiseless or error-corrected quantum hardware is available. Here, our utilized quantum circuit optimization approach [48] combined with learning-based error mitigation (EM) techniques [49] yielded comparable test performance in the quantum simplified SVM (qsSVM) approach to the noiseless simulator results in both 3 and 4 qubit configurations (8 and 16 features, respectively). In contrast, the quantum kernel Gaussian process (qGP) QML approach underperformed even when relying on EM (BACC: 69% in IonQ with EM, 73% on simulator). While the quantum distance classifier (qDC) yielded identical results with qsSVM with EM and in simulator (BACC: 73%), qDC did slightly underperform on IonQ without EM (BACC: 64%). The reasons of this are manifold: The qsSVM runs only once and with a so-called Swap-test circuit. In comparison, the qGP runs three-times with Swap-test, while the qDC runs once, but with the so-called Hadamard-test, requiring a more complex circuit [13, 20]. It is crucial to understand what tests can be combined with what QML algorithms when utilizing real quantum hardware. As such, swap test can result in information loss by the means of the sign of the train-test inner products that are required for the prediction [50]. Nevertheless, Swap-test can be combined with qsSVM and qGP because they operate with positive semi-definite kernel matrices [30]. In contrast, the qDC approach requires the sign of the inner products to be preserved, which can be achieved with Hadamard-tests that are in return, more complex. The relevance of quantum circuit complexity and well as the number of its measurements manifests in the noisy nature of existing quantum hardware, also referred to as noisy intermediate scale quantum systems (NISQ). This noise can degrade the output result of quantum circuits, affecting QML predictive performance. Therefore, when relying on NISQs, these effects need to be mitigated. As such, non-mitigated results in our study represent a wide-range of test BACCs (59–68%), while we successfully achieved the highest test BACC of 73% being identical with noiseless simulator results with the qsSVM and qDC algorithms relying on our mitigation techniques in the IonQ Aria device. In this regard, our research demonstrates that even when utilizing circuit optimization and error mitigation in combination with radiomic data, the understanding of what QML algorithms and what circuit tests shall be and can be utilized together is imperative.

Our study has multiple implications in the field of in vivo disease characterization, particularly when focusing on radiomic studies. First, relying on GD_Q_ estimations [14], future research can estimate whether it makes sense to approach the given radiomics task in the quantum computing domain (test if GD_Q_ > 1.0). Second, when a quantum advantage is anticipated, widely available simulator environments combined with appropriate kernel methods can yield superior predictive performances with QML compared to CML, which is especially emphasized with small data. Third, when engaging with real quantum hardware is an option, the high-fidelity of NISQs relying on appropriate classic-to-quantum data encoding results in simpler, robust, and potentially interpretable models due to encoding N radiomic features with log2*(N)* number of qubits. This can also support endeavors to combine shallow and deep radiomic as well as non-imaging features together [7] to potentially yield high-performing QML holistic models. In this case, our circuit optimization technique combined with error mitigation can support researchers to minimize noise in existing NISQs and potentially, to even achieve higher predictive performance compared to simulators in case high-fidelity NISQ devices become widely available.

This study had limitations. First, it only operated with single center cohorts; however, it was utilizing cross-validation to estimate the performance and to compare a wide-range of classic and quantum ML approaches and with different SRTs as well as feature counts. Second, due to restricted access to the IonQ device, the effects of circuit optimization and error mitigation could only be demonstrated with selected QML approaches and for one train-test setting, thus, in a hold-out validation scenario. Nevertheless, the essence of our findings in the context of utilizing NISQs in a feasible way was not related to this limitation. Last, while additional CML approaches could have been involved in our study, those either do not yet have a QML variant, of their QML variant — such as quantum random forests — is not designed to be executable on existing NISQs [51].

## Conclusions

We conclude that in case measurable conditions apply, quantum advantage can be demonstrated in clinically relevant cancer imaging cohorts relying on classic radiomic features and quantum machine learning. In the near-term, additional quantum machine learning predictive performance improvements may be achieved when relying on circuit optimization and error mitigation techniques in real quantum hardware, having much higher fidelity gates.

### Supplementary Information

Below is the link to the electronic supplementary material.

Supplementary file1 (PDF 1967 KB)

## Data Availability

Data is available from the respective corresponding authors of the studies this work relied on to perform its retrospective analysis. Data generated by this study is available from the corresponding author.

## Declarations

### Access

For accessing the source code of quantum machine learning executions this study relied on, refer to: https://github.com/sassan72/learning-with-Quantum-machines
