# Supplementary material for: Error mitigation enables PET radiomic cancer characterization on quantum computers
Source: Eur J Nucl Med Mol Imaging. 2023 Aug 4;50(13):3826–37. doi: 10.1007/s00259-023-06362-6 (PMC10611844; doi:10.1007/s00259-023-06362-6)
Supplement: Supplementary file 1 — Supplementary file1 (PDF 1967 KB) [file 259_2023_6362_MOESM1_ESM.pdf]

Supplementary information for “Error mitigation enables PET radiomic cancer characterization on quantum computers”

S. Moradi, C. Spielvogel, D. Krajnc, C. Brandner, S. Hillmich, R. Wille,
T. Traub-Weidinger, X. Li, M. Hacker, W. Drexler, and L. Papp*

(Dated: June 22, 2023)

* laszlo.papp@meduniwien.ac.at

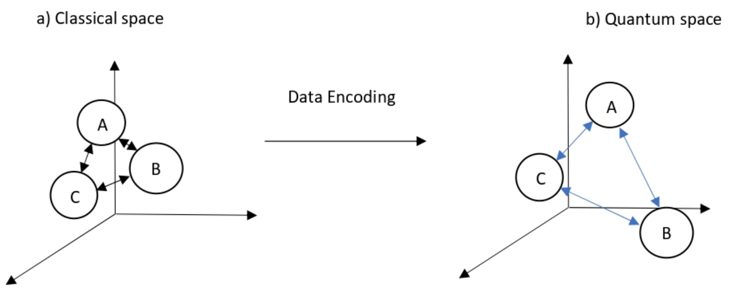

FIG. 1. a) The position of three different data points A, B, and C in the classical space. b) After encoding classical data into quantum Hilbert space, the kernel entries corresponds to the data points in the quantum space decrease (distances increase). In this case the geometry difference (GD_Q) score decreases.

The math and details are in support of the main text. The source code of the implemented classical and quantum algorithms can be accessed by the link:

Appendix A: Geometry difference and its physical meaning

In order to link amplitude data encoding, distance function, and the quantum kernel to the prediction advantage, the geometric difference GD_Q is used which only depends on the dataset, but is independent of the labels. The geometric difference measures the similarities of different distance functions and different kernel functions of the same dataset. The geometric difference machine learning models based on two different kernel functions K_Q (quantum kernel) and K_C (classical kernel) is defined by [1]

$$GD_Q = G(K_C||K_Q) = \sqrt{\|\sqrt{K_Q}(K_C)^{-1}\sqrt{K_Q}\|} \tag{A1}$$

where $\|.\|$ is the spectral norm of the resulting matrix. GD_Q defined in Eq. (A1) measures the difference between how a quantum ML vs classical ML sees the relation between data [1]. If $GD_Q \approx 1.$, then the prediction performance of quantum ML models and classical ML models are the same.

The physical meaning of GD_Q is explained as follows: a kernel matrix is a semi definite matrix whose diagonal entries are 1 and off-diagonal entries are less than 1. The off-diagonal entries of a kernel matrix can be seen as measures of distance between data points in the same feature space. Mathematically, the off-diagonal entry of a kernel matrix $K(x_i, x_j)$ is large when the distance between x_i and x_j is small. Fig. 1 shows the positions of three data points A, B, and C in classical and quantum feature spaces, respectively, with the arrows representing the distances between data points. After encoding data from classical Euclidean space into quantum Hilbert space, the distance between data points increases. From the kernel point of view, the entries of classical kernel matrix are larger compared to the entries of the quantum kernel matrix.

For example, if the classical kernel matrix corresponds to Fig. 1(a) is

$$K_C = \begin{bmatrix} 1.0 & 0.9 & 0.8 \\ 0.9 & 1.0 & 0.7 \\ 0.8 & 0.7 & 1.0 \end{bmatrix} \tag{A2}$$

and the quantum kernel matrix corresponds to Fig. 1(b)

$$K_Q = \begin{bmatrix} 1.0 & 0.3 & 0.3 \\ 0.3 & 1.0 & 0.2 \\ 0.3 & 0.2 & 1.0 \end{bmatrix} \tag{A3}$$

then the $GD_Q = 2.681$.

In the second case, if the classical kernel matrix corresponds to Fig. 2(a) is

$$K_C = \begin{bmatrix} 1.0 & 0.3 & 0.3 \\ 0.3 & 1.0 & 0.2 \\ 0.3 & 0.2 & 1.0 \end{bmatrix} \tag{A4}$$

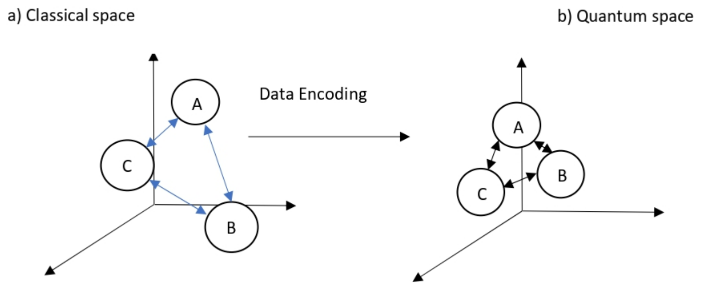

FIG. 2. The position of three different data points A, B, and C in the classical space. b) After encoding classical data into quantum Hilbert space, the kernel entries correspond to the data points in the quantum space increase (distances decrease). In this case GD_Q increases.

and the quantum kernel matrix corresponds to Fig. 2(b) is

$$K_Q = \begin{bmatrix} 1.0 & 0.9 & 0.8 \\ 0.9 & 1.0 & 0.7 \\ 0.8 & 0.7 & 1.0 \end{bmatrix} \tag{A5}$$

then the $GD_Q = 0.924$.

The first classical kernel that we used in this study was homogeneous linear kernel which is the simplest kernel function to compute. The linear covariance function is given

$$K_{\text{linear}}(x_1, x_2) = x_1^T x_2 \tag{A6}$$

The second kernel function is the Matern kernel. The Matern class of covariance function is given by

$$K_{\text{Matern}}(x_1, x_2) = \frac{2^{1-\nu}}{\Gamma(\nu)} \left(\frac{\sqrt{2\nu} r(x_1, x_2)}{l} \right)^{\nu} K_{\nu} \left(\frac{\sqrt{2\nu} r(x_1, x_2)}{l} \right) \tag{A7}$$

where $r^2(x_1, x_2) = (x_1 - x_2)^T (x_1 - x_2)$, ν and l are positive values. K_ν is a modified Bessel function. The Matern covariance function in Eq. (A7) becomes especially simple when ν is half integer: $\nu = p + \frac{1}{2}$ and p is a non-negative integer. In this case the covariance function is product of a exponential and a polynomial of order p [2]. The parameter ν controls the smoothness of the function. For smaller ν results in the less smooth kernel function. For $\nu = \inf$, the kernel function equivalent to the RBF kernel and $\nu = 0.5$ to the absolute exponential kernel. Other values for ν are $\frac{3}{2}$, and $\frac{5}{2}$, which are easy to compute. The computational cost are approximately 10 times higher for other values of ν in comparison with $\nu = 0.5, 1.5, 2.5$, and ∞, since they require to evaluate the modified Bessel function. In another numerical simulation, we chose $l = 1.0$ and $\nu = 3.0$ for the Matern kernel in Eq. (A7) such that the $GD_Q \approx 1$s for almost all 10-fold. The predictive performance for the classical ML algorithms cSVM and cGP were the same as the quantum ML algorithms qSVM and qGP.

Appendix B: Data encoding

Noticeable quantum speedup of many quantum machine learning algorithms for real-world datasets [3–5] are due to amplitude encoding, since N numerical features of the data vectors can be represented with $\log_2 N$ number of qubits of the quantum states in the quantum computer [6, 7]. The majority of researches for amplitude encoding with single-qubit and two-qubit gate operations rely on algebraic methods. Algebraic methods are guaranteed to work for any problem size of qubits, but are likely to result in quantum circuits that are exponentially large [8–11]. Another approach is via exploration of possible circuits topology in conjunction with classical optimization techniques [12, 13]. This approach which guarantees to find the smallest circuit for data encoding with few qubits.

Following we introduce two circuit optimization methods. The first method can be implemented with only quantum simulators, but the second with both quantum simulators and current NISQ devices. Both methods give the same results, if one uses the quantum simulator.

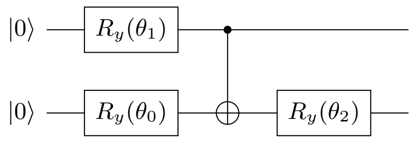

FIG. 3. Quantum circuit for mapping a four-dimensional state vectors to two qubit states by applying three single R_y rotation gates with rotation angles of θ_i with a $CNOT$ gate in between. θ_i are obtained using optimization of cost function given in Eq. (B2).

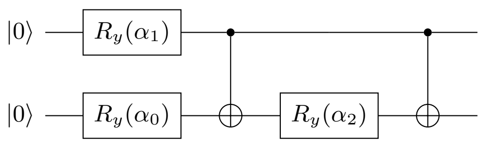

FIG. 4. Quantum circuit for mapping a four-dimensional state vectors to two qubits states by applying three single R_y rotation gates with two $CNOT$ gates. This quantum circuit relies on algebraic method. The rotation angles are obtained analytically.

a. Circuit optimization with noiseless quantum simulator

We start by describing our data encoding strategy via a numerical approach. Representing classical data as quantum states is an important step for QML applications, since most QML algorithms require efficient quantum access to classical data. A data encoder is a quantum circuit that, given a classical data vector $\vec{a} = (a_0, a_2, \ldots, a_{N-1}) \in R^N$, prepares quantum state

$$\frac{1}{\|a\|} \sum_{i=0}^{N-1} a_i |i\rangle. \tag{B1}$$

Eq. (B1) represents an amplitude encoding state. Fig. 3 shows the smallest quantum circuit for amplitude encoding of classical data with 4-feature counts. A quantum circuit for amplitude encoding with algebraic approach can also be seen in Fig. 4. See Ref. [14] for how to obtain the α_i rotation angles. In the next step, we use classical optimization methods to find the values for the θ_i rotation angles in Fig. 3 through minimizing of a cost function on a classical computer. The cost function is defined as [8]

$$C(V(\vec{\theta}), U) = \frac{1}{N^2} \sum_{j=0}^{N-1} \sum_{k=0}^{N-1} |V_{jk}(\vec{\theta}) - U_{jk}| \tag{B2}$$

where U_{jk} is jk^{th} element in the known unitary matrix U given by quantum circuit in Fig. 4 and $V_{jk}(\vec{\theta})$ is jk^{th} element in the target unitary matrix V given by quantum circuit in Fig. 3. The desired values for θ_i are computed when the right side of the Eq. (B2) converges to zero. Therefore, an exact quantum circuit for V can be found.

We now perform a structure update on previous quantum circuit for two qubits to find the quantum circuit for three qubits. This time two-qubit gates on the second and third qubits and the first and the third qubits are applied, as can be seen in Fig. 5. To obtain the values of the free parameters in Fig. 5, the same procedure for the two-qubit case is followed. Iterating this procedure then leads to a design for quantum circuits for larger number of qubits. This approach is called *fix structure strongly entangling layer*, since all qubits are entangled with each other using two-qubit gates. Fig. 6 shows the quantum circuit for amplitude data encoding with the algebraic method which includes six $CNOT$ gates.

Figs. 7, 8, and 9 show the cost function $C(V(\vec{\theta}), U)$ as a function of the number of iterations for $n = 2, 3$, and 4 qubits. The cost functions converge to the desired global minimum with the gradient-based, LBFGS, and Powell optimization methods after 100, 3, and 3 iterations, respectively.

Figs. 10 and 11 show the quantum circuits for data encoding with 4 and 5 qubits relying on algebraic method. As can be seen from Figs. 10, 11, fourteen $CNOT$s are applied to encode a vector with 16-components into a quantum state and thirty $CNOT$s in the 32-components case. In general $2^n - 2$ $CNOT$ gates and $2^n - 1$ single-qubit rotation R_y are applied to map 2^n-components vector onto n qubits quantum state given in Eq. (B1) based on algebraic method. While, with numerical approach, only six $CNOT$s are needed to encode 16-components into a quantum state (see Fig. 12) and ten $CNOT$s for 32-components. The shorter depth quantum circuits help to reduce the run time, and as well as the quantum noise of the QML algorithms on NISQ devices.

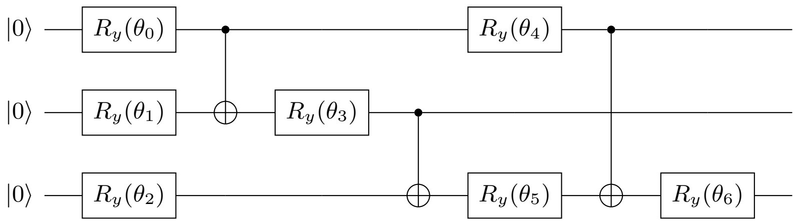

FIG. 5. Topology of the quantum circuit for encoding a eight-dimensional state vector to three-qubit states relying on numerical optimization. Seven single R_y rotation gates with three $CNOT$ gates are applied for amplitude data encoding.

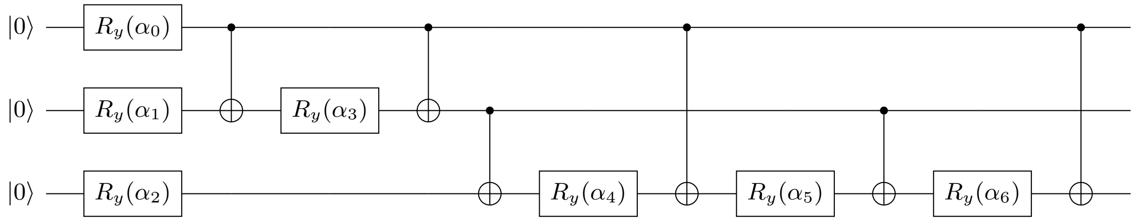

FIG. 6. Topology of the quantum circuit for encoding a eight-dimensional state vector to three-qubit states relying on algebraic approach. Seven single R_y rotation gates with six $CNOT$ gates are applied for amplitude data encoding.

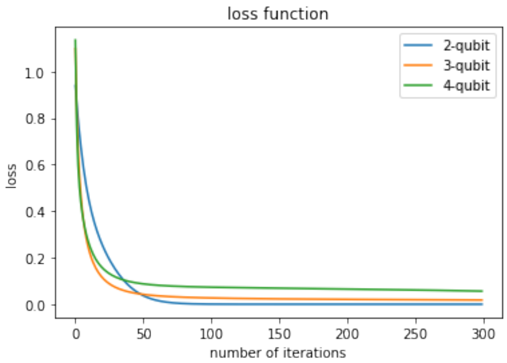

FIG. 7. Result of performing continuous parameter optimization using the gradient descent method. The minimum of the cost function is achieved after 100 iterations with the maximum cost error tolerance 10^{-2}.

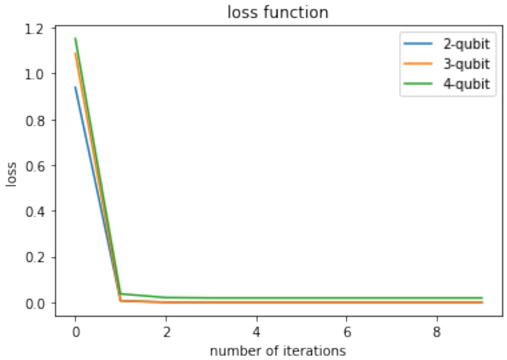

FIG. 8. Result of performing continuous parameter optimization using he LBFGS method. The minimum of the cost function is achieved after a maximum of 3 iterations for $n = 2, 3$, and 4 qubits with the maximum cost error tolerance 10^{-3}.

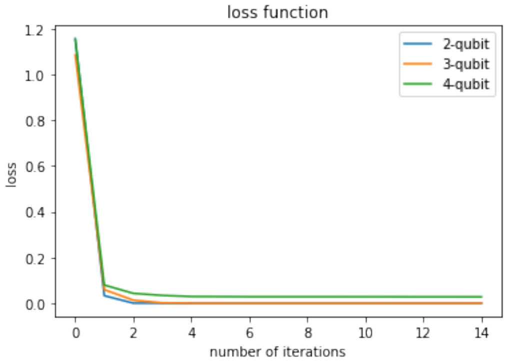

FIG. 9. Result of performing continuous parameter optimization using the Powell method. The minimum of the cost function is achieved after a maximum of 3 iterations for $n = 2, 3$, and 4 qubits with the maximum cost error tolerance 10^{-2}.

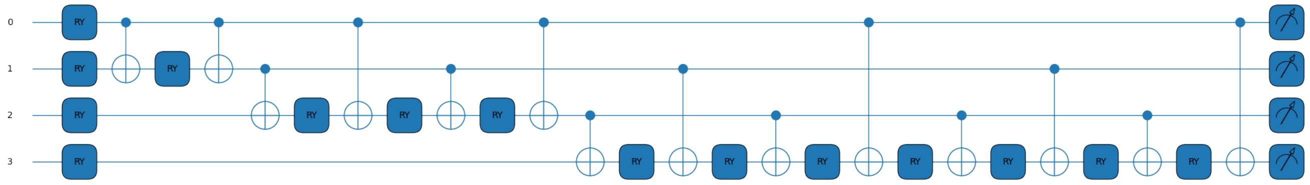

FIG. 10. Quantum circuit for preparing the quantum state $|\psi\rangle = \frac{1}{\|a\|}\sum_{i=0}^{15} a_i|i\rangle$. This quantum circuit can encode a vector with 16-components into a quantum state with four qubits.

b. Circuit optimization with NISQ devices

The cost function was defined in Eq. (B2) can not be implemented on NISQ devices, since the current NISQ devices can only estimate the expectation value of observables. The strategy is to define a new cost function C through evaluating the expectation values with a NISQ device, while a classical optimizer trains the parameters $\vec{\theta}$. For this purpose, we define a cost function which is a quadratic distance between U_T and parameterized unitary $U(\theta)$ [15]

$$C(\vec{\theta}) = 1 - |\langle 0|U_T U(\vec{\theta})|0\rangle|^2. \tag{B3}$$

We first estimate the term $|\langle 0|U_T U(\vec{\theta})|0\rangle|^2$ using a Swap Test circuit (see Fig. 13) on a NISQ device [12]. Following the cost function $C(\vec{\theta})$ on a classical computer using Eq. (B3) is calculated and the optimization approaches are applied. Each iteration for optimization can include several calls to the NISQ device. The vector $\vec{\theta}$ in the ansatz $U(\vec{\theta})$ is adjusted in a hybrid quantum-classical optimization loop until the cost $C(\vec{\theta})$ is converged below a threshold.

Appendix C: Gate Decomposition

In order to execute a quantum circuit on different NISQ architectures, two-qubit gates must satisfy the coupling constraints of the architecture [16]. Since NISQ devices commonly support only single-qubit and two-qubit gate

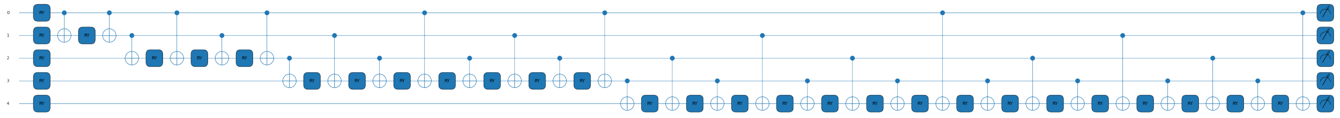

FIG. 11. Quantum circuit for preparing quantum the state $|\psi\rangle = \frac{1}{\|a\|}\sum_{i=0}^{31} a_i|i\rangle$. This quantum circuit can encode a vector with 32-components into a quantum state with five qubits.

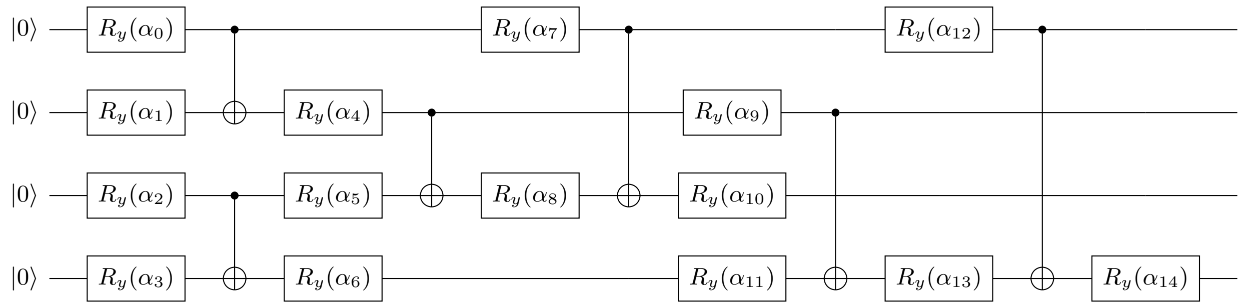

FIG. 12. Topology of the quantum circuit for mapping a sixteen-dimensional vectors to three qubits relying on numerical approach. Fifteen single R_y rotation gates with six $CNOT$ gates are applied for amplitude data encoding.

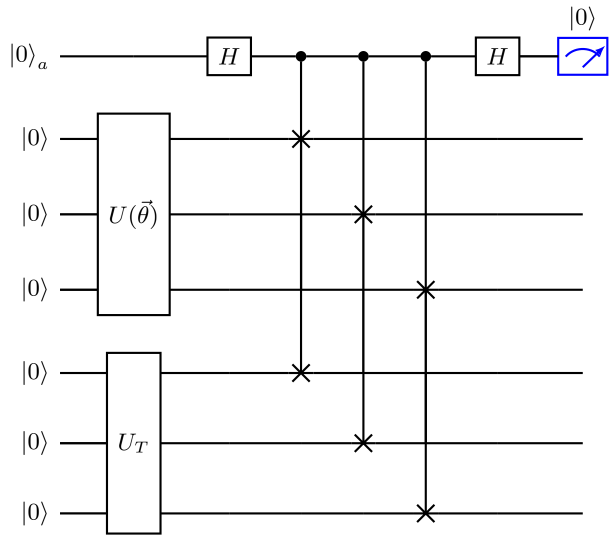

FIG. 13. Quantum circuit to compute $|\langle 0|U_T U(\vec{\theta})|0\rangle|^2$ for optimization of 3 qubits data encoding cases with NISQ devices. The target unitary U_T encodes the features of data-points via algebraic method into amplitudes of quantum states [10]. $U(\vec{\theta})$ is the parameterized circuit. The output result of the quantum circuit after measurement yields the value of $|\langle 0|U_T U(\vec{\theta})|0\rangle|^2$.

operations, complex gate operations must be decomposed into supported gates before mapping on noisy hardware. Owing to the specific architectures of different NISQ devices, two-qubit gate operations must satisfy the *coupling constraint* imposed by the coupling map of physical qubits [16], i.e., two-qubit gate operations can only be performed between certain pairs of qubits. For example, if q_i is the control qubit and q_j is the target qubit, $CNOT(q_i, q_j)$ can only be applied if there is coupling between q_i and q_j. Otherwise $CNOT(q_i, q_j)$ must be mapped into executable $CNOT$ gate operations. The compilation of quantum circuit for the Swap Test and the Hadamard Test on NISQ devices are computationally expensive due to existence of Toffoli and three-qubits controlled swap (Fredkin) gates (See Figs. 14, 15, and 16). To compile the quantum circuits for the Swap Test and the Hadamard Test on the 21-qubits IonQ quantum machine (see Appendix G), we decomposed the non-Clifford gates (Toffoli and Fredkin gates) to single-qubit and $CNOT$ gates. Each Fredkin gate is decomposed into two $CNOT$s and one Toffoli gate as Fig. 14 shows [17].

The next step is to decompose Toffoli gate in Fig. 14 with Margolus technique [17–19] and Qiskit [20]. The Toffoli gate requires six $CNOT$s with the transpilation technique of Qiskit as can be seen from Fig. 15, but three $CNOT$s with Margolus' technique (see Figure. 16).

where

$$G = \begin{bmatrix} \cos\frac{\pi}{8} & -\sin\frac{\pi}{8} \\ \sin\frac{\pi}{8} & \cos\frac{\pi}{8} \end{bmatrix}$$

and $G^\dagger$ is the hermitian conjugate of G.

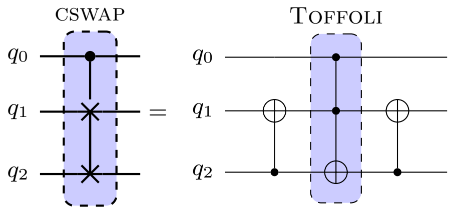

FIG. 14. Decomposition of $CSWAP(q_0, q_1, q_2)$ to two $CNOT$s and one Toffoli gate.

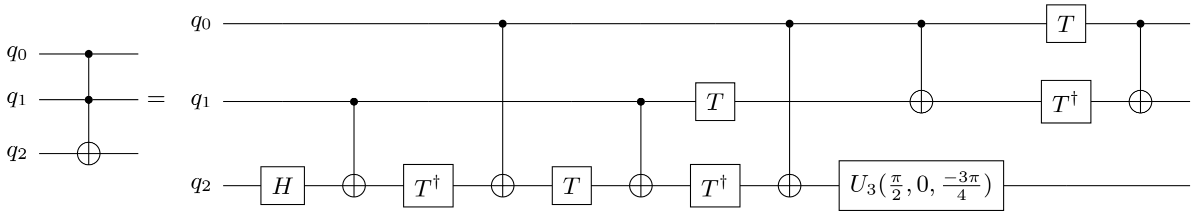

FIG. 15. Decomposition of Toffoli gate with Qiskit transpile function. Toffoli gate is decomposed to six $CNOT$ gates and eight single qubit gates.

The $CNOT$ gate can not be implemented directly on the IonQ quantum machine. The physical two-qubit gate available to the IonQ quantum machine is the $R_{xx}(\xi)$ gate [21], defined as follow:

$$R_{xx}(\xi) = \begin{bmatrix} \cos\xi & 0 & 0 & -i\sin\xi \\ 0 & \cos\xi & -i\sin\xi & 0 \\ 0 & -i\sin\xi & \cos\xi & 0 \\ -i\sin\xi & 0 & 0 & \cos\xi \end{bmatrix}$$

The parameter ξ depends on the pair of ions the gate is being applied. Fig. 17 shows the equivalent quantum circuit for $CNOT$ with $\xi = \frac{\pi}{4}$.

Another gate operation which is very important and must be decomposed before mapping Hadamard test circuit (see Fig. 23) on the 21-qubits IonQ quantum machine is single control rotation y rotation $CR_y(\theta)$ gate. Using the Qiskit transpile function, each $CR_y(\theta)$ gates can be decomposed into two single qubit y rotation gate $R_y(\frac{\theta}{2})$ and two $CNOT$ as Fig. 18 shows.

Appendix D: Quantum machine learning algorithms for binary classification

1. Quantum distance classifier

First, we divide the train set, with M number of samples, based on their labels $\{a, b \in R\}$ into two subset $\{P\}$ and $\{Q\}$, where $\{P\}$ contains only label a with the number of samples MP and $\{Q\}$ contains only label b with the number of samples MQ with $MP + MQ = M$. In the quantum distance classifier (qDC), first, the distance is calculated between the state vector of a test sample and each state vector of the train sample in set P and set Q and, then, assigns a label of the test sample to the label of the closest set. The task is to determine the label of the given test sample y_k, if $y_k = a$ or $y_k = b$. Mathematically, if $|v\rangle$ is the state vector of the test sample as well as $|u\rangle \in P$ and

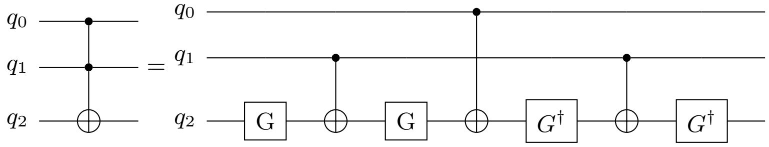

FIG. 16. Decomposition of Toffoli gate to three $CNOT$s and four single qubit gates with Margolus technique.

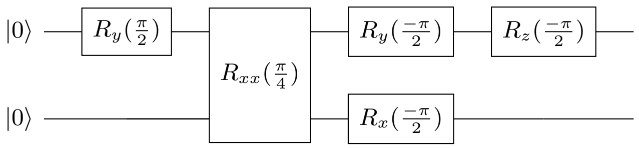

FIG. 17. Decomposition of $CNOT$ to two qubit Mølmer–Sørensen gate $R_{xx}(\xi)$.

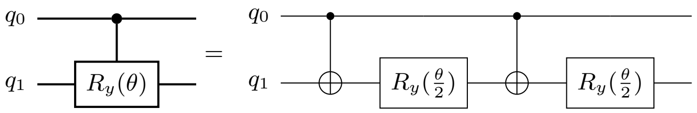

FIG. 18. Decomposition of $CR_y(q_0, q_1)$ to two $CNOT$s and two R_y gates with half angle rotations.

$|w\rangle \in Q$, then the label of $|v\rangle$ is determined by $y_k = a$, if $\min d(|u\rangle - |v\rangle) \leq \min d(|w\rangle - |v\rangle)$, otherwise $y_k = b$. The distance between vectors is given by [22]

$$d(|u\rangle - |v\rangle) = \||u\rangle\|\||v\rangle\| - \langle u|v\rangle \tag{D1}$$

where $\|.\|$ is the norm l_2 of a vector in Eq. (D1) where $\||u\rangle\| = 1$ and $\||v\rangle\| = 1$. Therefore, the task is to calculate the inner product $\langle u|v\rangle$ with a NISQ device.

2. Quantum kernel Gaussian Process

Consider a binary classification problem with a train dataset which we call D such that

$$D = (\vec{X}, y) = \{\vec{x}_i, y_i\}_{i=0\cdots M-1}\,, \tag{D2}$$

where $\vec{x}_i$ is an N-dimensional vector and labels $y_i \in \{-1, 1\}$. The task is to model a function which can generate the labels within a dataset from given input vectors such that

$$y = f(\vec{X}) + \epsilon_{noise}, \tag{D3}$$

where $\epsilon_{noise} \sim \eta(0, \sigma^2)$ represents a distributed Gaussian noise with zero mean and variance σ^2. The function of the Gaussian process algorithm is to predict an unknown label y_* for a given test data vector $\vec{x}_*$. A scalar Gaussian Process (GP) is defined as the multivariate normal distribution $GP(m(.), k(., .))$, where $m(.)$ is a mean function and $k(., .)$ is a covariance function. The joint distribution of y as the label of the train data and y_* as the unknown label of $N-$dimensional vector test $\vec{x}_*$ is defined

$$y_*|(\vec{X}, \vec{x}_*, \epsilon^2_{noise}, y) \sim GP(m, k), \tag{D4}$$

where $m = K_*^T(K + \Sigma)^{-1}y$, $k = K_{**} + \Sigma - K_*^T(K + \Sigma)^{-1}K_*$, and $\Sigma = \sigma^2 I$ is a $M \times M$ diagonal matrix. K is the train kernel matrix, K_* is the train-test kernel matrix, K_*^T is the test-train kernel matrix, and K_{**} is the test kernel matrix. In the binary classification, the joint probability for y_* to be 1 at $\vec{x}_*$ given train dataset $\vec{X}$ and label y can be approximated by [23]

$$p(y_* = 1|y, \vec{X}, \vec{x}_*) \approx S(\kappa m), \tag{D5}$$

where $S(x) = \frac{1}{1+e^{-x}}$, a sigmoid function, and $\kappa = \frac{1}{\sqrt{1+\frac{\pi k}{8}}}$. Tresholding the value $S(\kappa m)$ yields the binary output as following

$$y_* = \begin{cases} 1 & \text{if } S(\kappa m) \geq 0.5 \\ -1 & \text{otherwise} \end{cases}\,. \tag{D6}$$

For the qkGP algorithm, the NISQ device is used three times to estimate K, K_*, and K_{**} (see Fig. 19).

3. Quantum kernel machine learning without optimization

For the quantum kernel SVM without optimization (qsSVM) [22], the standard form of the kernelized binary classifier is

$$y_* = sgn\Big(\sum_{i=1}^{M} y_i \alpha_i^* K(\vec{x_i}, \vec{x}^*)\Big) \tag{D7}$$

where y_* is the unknown label of the test dataset $\vec{x}^*$, y_i is the label of the ith train sample, α_i^* is the ith component of the support vector $\vec{\alpha}^* = (\alpha_1^*, \alpha_2^*, ..., \alpha_M^*)$, M is the number of train data, and $K(\vec{x_i}, \vec{x}^*)$ is the kernel of the train-test pairs.
For a given dataset

$$D = (\vec{X}, y) = \{\vec{x}_i, y_i\}_{i=0 \cdots M-1}\,, \tag{D8}$$

where $\vec{x}_i$ is an $N-$dimensional vector and labels $y_i \in \{-1, 1\}$, one option to calculate the support vector $\vec{\alpha}^*$ without optimization of complex function as presented in [22] is to set uniform weight $\alpha_i^* = 1$, in case of balanced dataset, $IR = 0.5$, where $IR = \frac{a}{b}$ with a being the number of minority class and b being the total number of samples. Otherwise, $\alpha_i^* = IR$ for the majority class and $\alpha_j^* = 1 - IR$ for the minority class applies. Thresholding the value $y_i \alpha_i^* K(\vec{x_i}, \vec{x}^*)$ yields the binary output

$$y_* = \begin{cases} 1 & \text{if } \sum_{i=1}^{M} y_i \alpha_i^* K(\vec{x_i}, \vec{x}^*) \geq 0 \\ -1 & \text{otherwise} \end{cases}\,. \tag{D9}$$

Fig. 20 represents a flowchart of the quantum kernel SVM (qkSVM) algorithm without optimization. NISQ device is used only once to estimate the kernel matrix of the train-test pairs.

Appendix E: Quantum neural network

The most basic and key ingredients of quantum neural network models are parameterized quantum circuits. In general, a quantum neural network (QNN) approach is the approximation of a complex function by another parameterized function $f(\vec{w}, b)$, which might be easier to compute with quantum circuits. The task is to find real parameters $\vec{w}$ and b which are expected to minimize a specific loss function $l(\vec{w}, b; D)$ for a given training dataset $D = \{(\vec{x_i}, y_i) : \vec{x_i} \in R^M, y_i \in \{-1, 1\}\}_{i=0,...,M-1}$. The goal of the QNN (a.k.a VQC) is to reduce the time complexity of the evaluation of the cost function $l(\vec{w}, b; D)$ and to improve the performance of classification in some cases compared with its classical counterpart, the Classical Neural Network (CNN). We follow a QNN method very similar to [24]. The QNN protocol is composed of five steps. First, the classical train data are encoded into n qubits quantum state vectors by applying a sequence of unitary gate operations on $|0\rangle^{\otimes n}$ which will result in $U(\vec{x})\,|0\rangle^{\otimes n} = |\psi(\vec{x})\rangle$. Second, parameterized circuit $U(\vec{w})$ is applied to $|\psi(\vec{x})\rangle$. The result is the final state $U(\vec{w})\,|\psi(\vec{x})\rangle = |\psi(\vec{x}, \vec{w})\rangle$. Third, the probability of the measurement of the first qubit q_0 on the basis of $|1\rangle$ of n qubits quantum state $|\psi(\vec{x}, \vec{w})\rangle$ yields

$$P(q_0 = 1|\vec{x}, \vec{w}) = |\langle 1|\psi(\vec{x}, \vec{w})\rangle|^2. \tag{E1}$$

Eq. (E1) can also be written down as the expectation value of the measurement operator σ_z applied to the first of n qubits using Dirac notation,

$$P(q_0 = 1|\vec{x}, \vec{w}) = \frac{E(\sigma_z)}{2} + \frac{1}{2} \tag{E2}$$

and

$$E(\sigma_z) = \langle U^\dagger(\vec{w})\psi(\vec{x})|(\sigma_z \otimes I \otimes ... \otimes I)U(\vec{w})|\psi(\vec{x})\rangle \tag{E3}$$

where $U(\vec{w})$ is a quantum circuit model includes $CNOT$ and general single-qubit rotation gate

$$U(\vec{w}) = U(\phi, \theta, \omega) = R_z(\omega)R_y(\theta)R_z(\phi) = \begin{bmatrix} e^{\frac{-i(\omega+\phi)}{2}}\cos(\frac{\theta}{2}) & -e^{\frac{-i(\phi-\omega)}{2}}\sin(\frac{\alpha}{2}) \\ e^{\frac{-i(\phi-\omega)}{2}}\sin(\frac{\theta}{2}) & e^{\frac{i(\omega+\phi)}{2}}\cos(\frac{\theta}{2}) \end{bmatrix} \tag{E4}$$

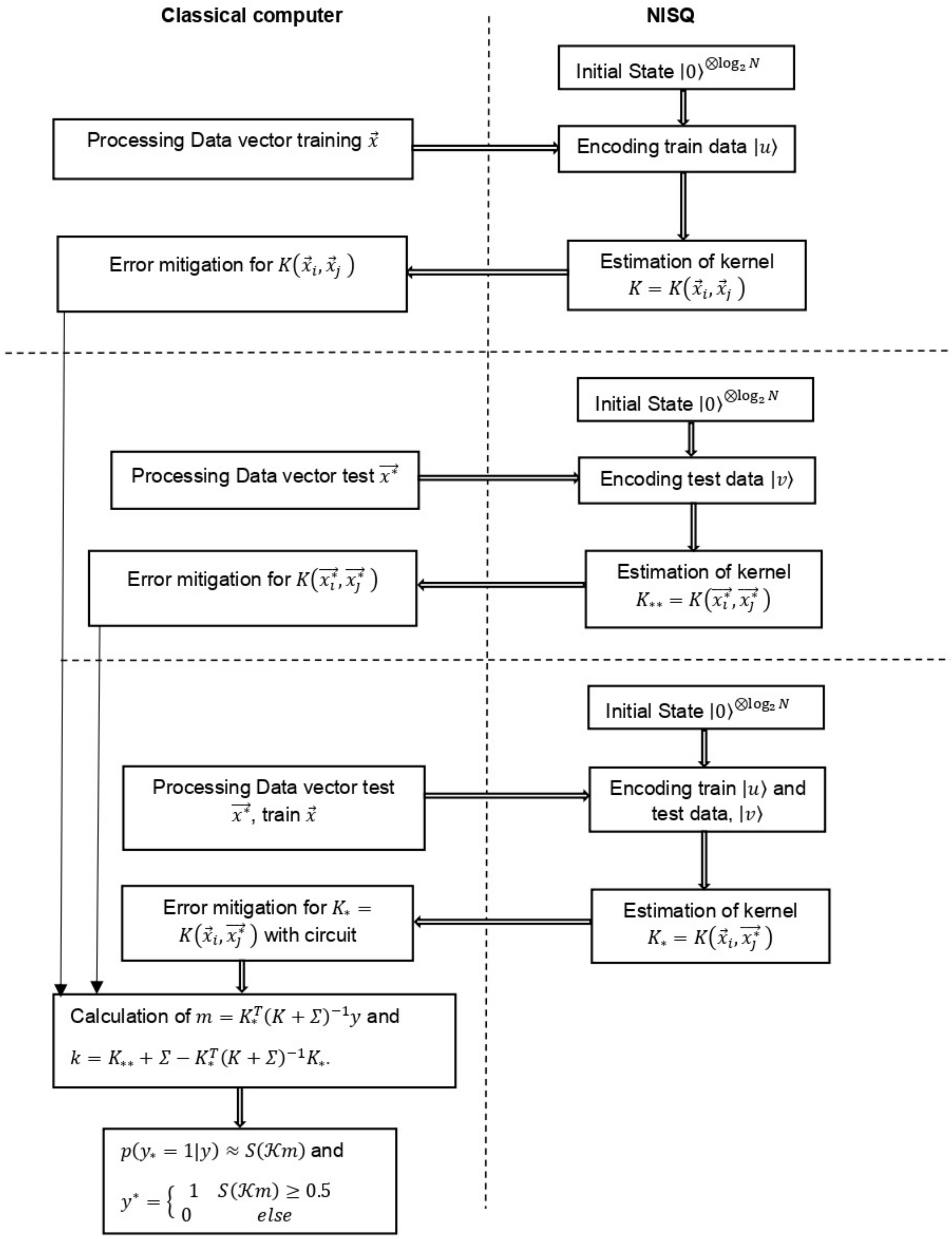

FIG. 19. Schematic of the Gaussian Process algorithm for binary classification. NISQ device is used three times to estimate $K(\vec{x_i}, \vec{x_j})$, $K(\vec{x_i^*}, \vec{x_j^*})$, and $K(\vec{x_i}, \vec{x_j^*})$. After estimation of kernel matrix in each step of the flowchart, error mitigation technique proposed in Appendix H is used to degrade the effects of noise and errors.

Like CNN, a learnable parameter is added to Eq. (E2) as bias term b,

$$P(q_0 = 1|\vec{x}, \vec{w}) = \left(\frac{E(\sigma_z)}{2} + \frac{1}{2}\right) + b \tag{E5}$$

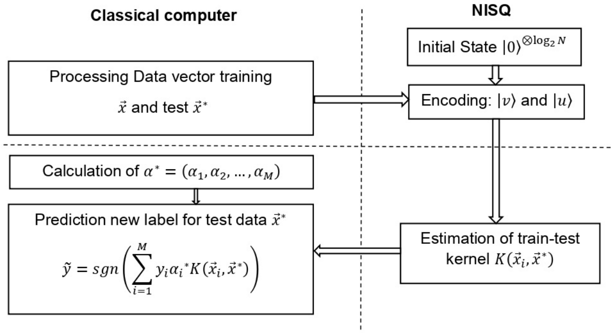

FIG. 20. Schematic of the qkSVM for binary data classification. First, the train data vector $\vec{x_i}$ and the test data vector $\vec{x}^*$ are prepared on a classical computer. Next, the train data and the test data data are encoded into quantum states $|u\rangle$ and $|v\rangle$ followed by computing the kernel matrix for all pairs of the train-test data $K(\vec{x}, \vec{x}^*)$ with a NISQ device. Then the error mitigation technique is utilized to reduce the effects of noise and errors on the NISQ device. If $\vec{\alpha}^* = (\alpha_1^*, \alpha_2^*, ..., \alpha_M^*)$ are considered to be a solution of the support vector, the binary classifier can be constructed based on Eq. (D3).

In step 4, the loss function given by $l(\vec{w}, b; D)$ is minimized

$$l(\vec{w}, b; D) = \sum_{i=0}^{M-1} |P(\vec{x_i}, \vec{w}, b) - y_i|^2 \tag{E6}$$

After finding the optimal parameters $\vec{w}, b$, a binary classifier can be constructed based on Eq. (E5). To find the label of the test vector $\vec{x_t}$,

$$y_* = \begin{cases} \;\;1 & \text{if } P(\vec{x_t}, \vec{w}_{opt}, b_{opt}) \geq 0.5(1-b) \\ -1 & \text{otherwise} \end{cases} . \tag{E7}$$

where $\vec{w}_{opt}$, and b_{opt} are the solution to the minimization of loss function of Eq. (E6). In order to minimize Eq. (E6), gradient descent method is used to update parameters, for the given dataset D. Fig. 21 shows a flowchart of the quantum neural network (QNN).

Appendix F: CML and QML hyper-parameters

The values of the hyper-parameters is documented in Tables I to VI.

All non-specified hyper-parameters were kept on the default values as specified by the official scikit-learn documentation (`https://scikit-learn.org/`).

For QML algorithms, the hyper-parameters for quantum kernel ML methods were considered the same as for classical kernel support vector machine.

For the quantum neural network classifier, we utilized the same parameters as we used for classical neural network with Adam optimizer. The only different is that instead of using activation function (ReLu function) in classical case, quantum measurement gate is applied for the QNN. The quantum distance classifier and quantum kernel Gaussian process are non-parameterized algorithms.

Appendix G: Software and hardware

For classical machine learning algorithms we use scikit-learn [25]. Qiskit [20] was used for experimenting with quantum circuits and Pennylane [26] for simulating them. We utilize Pennylane in combination with Pytorch for circuit optimization purpose. For the estimation of the kernel matrices and the inner products with the Swap Test and Hadamard Test on NISQ device, we choose the 21-qubit IonQ quantum machine. The gate-based IonQ quantum machine was available for this study as cloud computing services, hosted by Microsoft Azure [27]. Fig. 22 shows the coupling maps of the 21-qubit IonQ quantum machine. Each circle represents a trapped ion qubit. The lines in-between the qubits represent physical connections for implementation of two qubit gate. The single-qubit and two-qubit gate error rates can also be seen for the IonQ quantum machine and also via https://IonQ.com/technology.

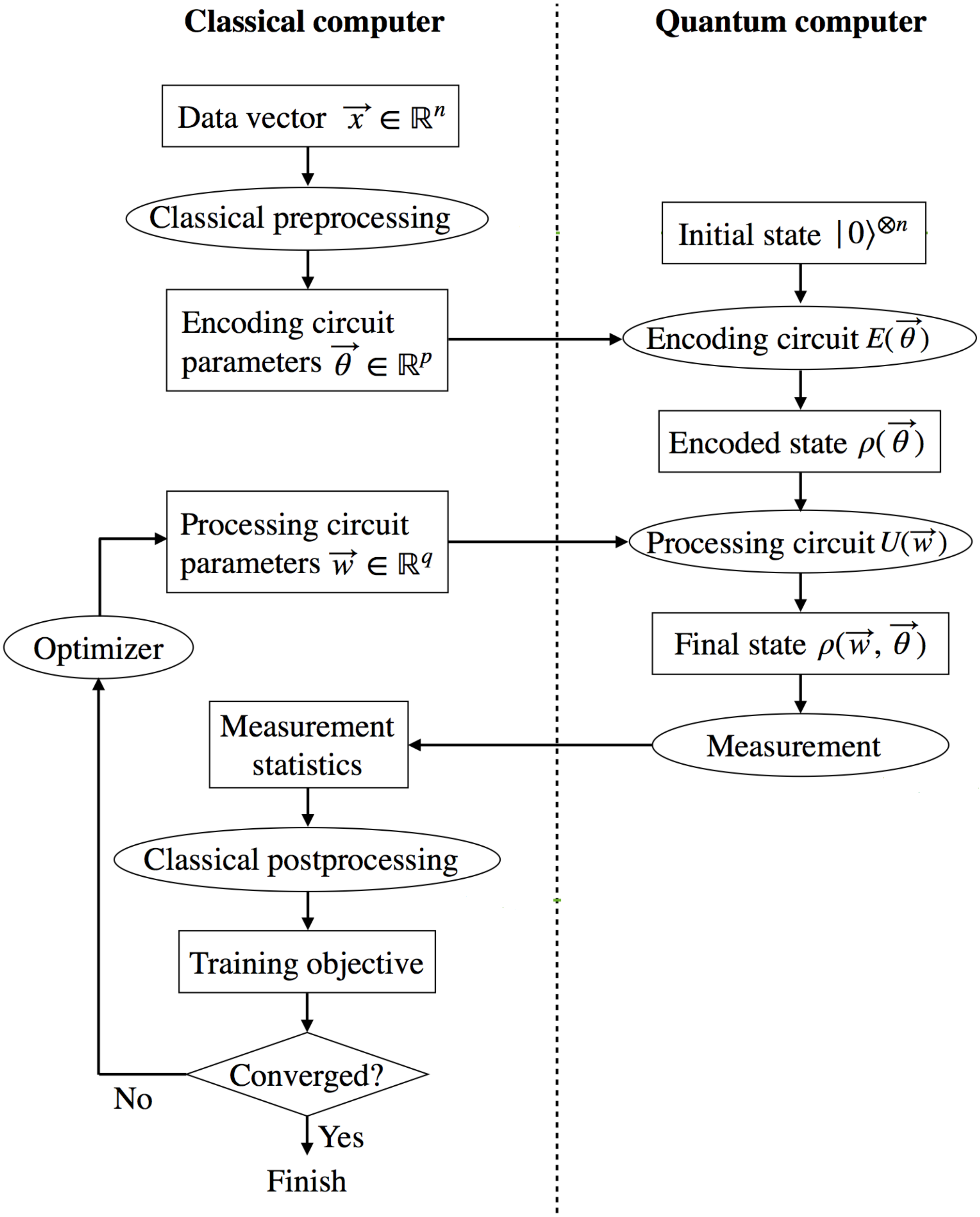

FIG. 21. Input data vectors are converted to circuit parameters. Then classical data is encoded into amplitudes of quantum states using a quantum circuit. Quantum measurement statistics are collected by repeatedly running the quantum circuit and performing measurements. Extracted information from the measurement statistics are obtained and the output label of the quantum classifier are produced by adding a bias term b (classical postprocessing). The loss function obtained from Eq. (E6) is minimized using Adam optimizer to yield the optimal parameters.

Random Forest for regression	
Hyper-parameters	Value
number of estimators	100
criterion	"gini"
max depth	None
min samples split	2
max features	"auto"
min samples leaf	1

TABLE I. Hyper-parameters for random forest regression algorithm. This algorithm was utilized for learning-based error mitigation.

k-nearest neighbors	
Hyper-parameters	Value
number of neighbours	5
weights	"distance"
p	2
number of jobs	-1

TABLE II. Hyper-parameters for classical k-nearest neighbors.

classical kernel support vector machine	
Hyper-parameters	Value
penalty	"l2"
loss	"squared hinge"

TABLE III. Hyper-parameters for classical kernel support vector machine.

classical neural network	
Hyper-parameters	Value
hidden layer sizes	(100,)
activation	"relu"
solver	"adam"
alpha	0.0001
batch size	"auto"
learning rate	"constant"
learning rate initial	0.001
max iteration	200
shuffle	true
tol	0.0001
early stopping	Flase
validation fraction	0.1
beta 1	0.9
beta 2	0.999
epsilon	$1e^{-8}$

TABLE IV. Hyper-parameters for classical neural network.

quantum kernel support vector machine	
Hyper-parameters	Value
penalty	"l2"
loss	"squared hinge"

TABLE V. Hyper-parameters for quantum kernel support vector machine with optimization.

quantum kernel support vector machine	
Hyper-parameters	Value
imbalance ratio	0.5

TABLE VI. Hyper-parameters for quantum kernel support vector machine without optimization.

quantum kernel support vector machine	
Hyper-parameters	Value
length scale	1.0
nu	3.0

TABLE VII. parameters for classical Matern kernel.

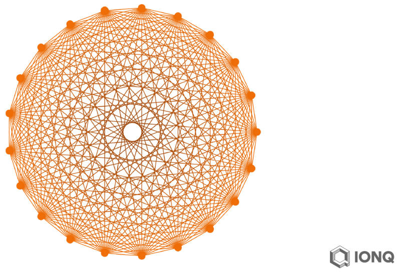

FIG. 22. Topology graph and coupling map of the 21-qubits IonQ quantum machine [29].

The elementary gates used on the IonQ quantum machine are single qubit gates and maximally entangling two qubit Mølmer–Sørensen gate (see Fig. 17 in Appendix B for decomposition of *CNOT* to Mølmer–Sørensen (R_{xx})). For the IonQ quantum machine, physical qubits are implemented as rare earth Ytterbium-171 ions ($^{171}\mathrm{Yb}^{+}$) trapped by electric fields and manipulated with a mode-locked 355nm laser which drives gate operations. The initial state of each $^{171}\mathrm{Yb}^{+}$ qubit has a coherence time of $T_1 \approx 10^7 \mu s$, $T_2 \approx 200,000 \mu s$.

The estimation of the kernel matrices and the inner product were done by measuring the state of an ancilla qubit. To do this with the IonQ quantum machine two species of ions are chosen: one for measuring the ancilla qubit and one for the other qubits that are not measured [28]. Reading the ion as an ancilla qubit is done by shining a resonant laser with a wavelength of 369.5nm such that the photon emitted by the ancilla qubit will not excite the other qubits [28].

Appendix H: Error mitigation strategy

Noise and errors are significant issues for capability of the current NISQ devices. Two important types of noise and errors in NISQ devices are gate errors and readout errors. The gate errors mostly result from miscalibration or imperfection in the control hardware and their interaction with the qubits. The gate errors include incoherent and coherent errors. Coherent errors are dominant gate errors [30]. Here we utilized a method by which the proper error mitigation strategy can instead be learned ab initio. On the contrary to other error mitigation methods, learning-based regression methods for error mitigation do not depend on the noise model of NISQ devices [31]. The readout errors concern the measuring of incorrect qubit values e.g. reading zero while the qubit is in the one state and vice versa.

First, the train dataset $\{x^{noisy}, x^{ideal}\}$ via executions of a Swap Test circuit or a Hadamard Test circuit (see Figs. 23 and 24 in Appendices I and J) on NISQ device $\{x^{noisy}\}$ and noiseless simulator $\{x^{ideal}\}$ were obtained. Then the Random Forest regression algorithm was trained with the train dataset $\{x^{noisy}, x^{ideal}\}$ to learn the relationship between x^{ideal} and x^{noisy}

$$x^{ideal} = f(x^{noisy}). \tag{H1}$$

Now Assume that we are given new $\{x_t^{noisy}\}$ values that has been obtained from the same Swap Test circuit (or the same Hadamard Test) on the NISQ device. Using Eq. (H1) and the new noisy values $\{x_t^{noisy}\}$ as input, the mitigated values are extrapolated $x_m^{noisy} = f(x_t^{noisy})$.

Appendix I: Estimation of the inner product

Fig. 23 shows the quantum circuit for estimation of the real part of $\langle u|v\rangle$ with the Hadamard Test. To estimate the real part of $\langle u|v\rangle$ on the quantum computer with the Hadamard Test, the train and test data needs to be prepared in

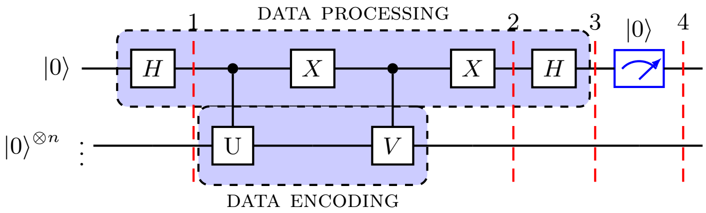

FIG. 23. Quantum circuit computes the real part of the inner product. Data processing step includes superposition, entanglement, and interference. The Hadamard gate puts the ancilla qubit $|0\rangle_a$ into uniform superposition. A single-controlled unitary gate entangles the exited state of the ancilla qubit with the train data state vector ($|u\rangle) = U\,|00\ldots0\rangle$). Another single unitary controlled gate entangles the state of the test data ($|v\rangle) = V\,|00\ldots0\rangle$) with the excited state of the ancilla qubit. A second X gate flips the ancilla qubit. The Hadamard gate on the ancilla qubit interferences train and test data state vectors. The ancilla qubit is measured on the $|0\rangle_a$ basis to estimate the value of $|\langle u|v\rangle|$ from Eq. (I3).

a quantum state as

$$\frac{1}{\sqrt{2}}(|0\rangle_a|u\rangle + |1\rangle_a|v\rangle) \tag{I1}$$

where $|u\rangle$ and $|v\rangle$ are the quantum states for the train and test datasets, respectively. Then the Hadamard gate on the ancilla qubit interferences the train vector $|u\rangle$ with the test vector

$$\frac{1}{2}\big(|0\rangle_a(|u\rangle + |v\rangle) + |1\rangle_a(|u\rangle - |v\rangle)\big). \tag{I2}$$

Finally, the measuring quantum state given in Eq. (I2) in the computational basis $|0\rangle_a$ gives

$$\Pr(|0\rangle_a) = \frac{1 + \langle u|v\rangle}{2}, \tag{I3}$$

where $\Pr(|0\rangle_a)$ is the value of the probability of measurement on the $|0\rangle_a$ state of Eq. (I2) and $\langle u|u\rangle = \langle v|v\rangle = 1$. Since our datasets are real, the NISQ device was used only once to calculate $\langle u|v\rangle$.

Appendix J: Estimation of the kernels with the Swap Test

Fig. 24 shows the quantum circuit for estimating $|\langle u|v\rangle|^2$ with a Swap test. First the quantum circuit encoder are applied (see Appendix B) to encode train and test data into quantum states $|u\rangle = U|00\ldots0\rangle$ and $|v\rangle = V|00\ldots0\rangle$, respectively. The Hadamard gate is applied on to the ancillary qubit $|0\rangle_a$ to create a superposition of $|u\rangle|v\rangle$, i.e.,

$$\frac{1}{\sqrt{2}}(|0\rangle_a|u\rangle|v\rangle + |1\rangle_a|u\rangle|v\rangle) \tag{J1}$$

The application of the single controlled-swap gates on the state given in Eq. (J1) generates an entangled state $\frac{1}{\sqrt{2}}(|0\rangle_a|u\rangle|v\rangle + |1\rangle_a|v\rangle|u\rangle)$. Then, another Hadamard gate is used to interfere the product state of the state vectors for train and test such that

$$\frac{1}{2}\big(|0\rangle_a(|u\rangle|v\rangle + |v\rangle|u\rangle) + |1\rangle_a((|u\rangle|v\rangle - |v\rangle|u\rangle)\big). \tag{J2}$$

The quantum state given in Eq. (J2) is measured in the computational basis of the $|0\rangle_a$ state to yield the probability

$$\Pr(|0\rangle_a) = \frac{1 + |\langle u|v\rangle|^2}{2}, \tag{J3}$$

where $\Pr(|0\rangle_a)$ is the probability of measurement on the $|0\rangle_a$ state of Eq. (J2).

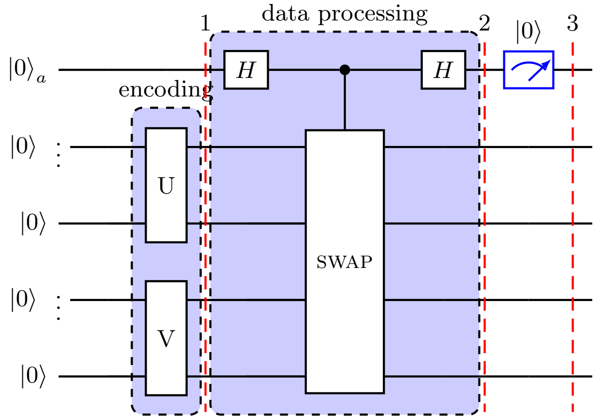

FIG. 24. Quantum circuit to compute kernels. 1, 2, and 3 satnd for data encoding, data processing, and measurement, respectively. Data processing step includes superposition, entanglement, and interference. The models of quantum circuits U and V encode data into amplitudes of quantum states $|u\rangle(= U|00\ldots0\rangle)$ and $|v\rangle(= V|00\ldots0\rangle)$. The quantum state of Eq. (J2) is measured on the $|0\rangle_a$ basis to estimate the value of $|\langle u|v\rangle|^2$ from Eq. (J3).

Appendix K: CLAIM: Checklist for Artificial Intelligence in Medical Imaging

In order to aid authors of AI manuscript, a checklist for AI in Medical Imaging has been proposed in this reference [32].

Section/ Topic	No.	Item	
TITLE/ ABSTRACT			
	1	Identification as a study of AI methodology, specifying the category of technology used (e.g., deep learning)	**Classic and quantum machine learning (ML) for predicting clinical endpoints from PET radiomics data.**
	**2**	Structured summary of study design, methods, results, and conclusions	**See ”Abstract” in manuscript.**
INTRODUCTION			
	**3**	Scientific and clinical background, including the intended use and clinical role of the AI approach	**Predicting clinical endpoints from 121 PSMA-11 prostate with low-vs-high Gleason risk, 84 11C-Methionine (MET) glioma with 3-years survival, and 335 18F-FDG PET lung cases with 2-years survival.**
	**4**	Study objectives and hypotheses	**Quantum ML in combination with error mitigation techniques makes clinical endpoint prediction from various cancer cohorts based on PET radiomics feasible on real quantum computers.**
METHODS			
Study Design	**5**	Prospective or retrospective study	**Prostate: Pre-study of a prospective clinical trial NCT02659527. Glioma and lung: retrospective.**
	**6**	Study goal, such as model creation, exploratory study, feasibility study, non-inferiority trial	**Feasibility and comparison study.**
Data	**7**	Data sources	**Open-access:**<br>**Prostate: https://osf.io/3nkx8/,**<br>**Glioma: https://osf.io/nkhp8/,**<br>**Access upon request: Lung**
	**8**	Eligibility criteria: how, where, and when potentially eligible participants or studies were identified (e.g., symptoms, results from previous tests, inclusion in registry, patient-care setting, location, dates)	**See CONSORT (Figure 1).**<br>**See DOI: 10.1007/s00259-020-05140-y,**<br>**DOI: 10.2967/jnumed.117.202267,**<br>**DOI: 10.1007/s00330-022-08999-7**

	**9**	Data pre-processing steps	**See "Feature ranking and selection" in manuscript.**
	**10**	Selection of data subsets, if applicable	**NA**
	**11**	Definitions of data elements, with references to Common Data Elements	**Prostate: Gleason risk "high" vs "low" mapped to 1 and 0 respectively.<br>Glioma: survived 3-years yes/no mapped to 1/0 respectively.<br>Lung: survived 2-years yes/no mapped to 1/0 respectively. See "Dataset" in manuscript for details.**
	**12**	De-identification methods	**Imaging data was anonymized upon downloading it from PACS via the in-built PACS anonymizer (Hermes Hybrid 3D ver. 4.0.0.)**
	**13**	How missing data were handled	**No missing data was present.**
Ground Truth	**14**	Definition of ground truth reference standard, in sufficient detail to allow replication	**See 11.**
	**15**	Rationale for choosing the reference standard (if alternatives exist)	**Clinical relevance.**
	**16**	Source of ground-truth annotations; qualifications and preparation of annotators	**Prostate: Full-mount histopathology slices from patients underwent radical prostatectomy. Glioma and lung: follow-up. See "Dataset" in manuscript for details.**
	**17**	Annotation tools	**Hermes Hybrid 3D ver. 4.0.0 to delineate and annotate lesions.**
	**18**	Measurement of inter- and intrarater variability; methods to mitigate variability and/or resolve discrepancies	**Multiple nuclear medicine physicians involved in delineation.<br>See DOI: 10.1007/s00259-020-05140-y,<br>10.2967/jnumed.117.202267,<br>10.2967/jnumed.117.202267 for details.**
Data Partitions	**19**	Intended sample size and how it was determined	**Sample size was given according to prior studies providing the datasets. Feature count was fixed to n=8 features and n=16 features to address ML curse of dimensionality issues. Three different Spearman rank 0.7, 0.8, and 0.9 were considered for this study.**
	**20**	How data were assigned to partitions; specify proportions	**Random subsampling of train (80%) and test (20%) samples in a 10-fold cross-validation scheme. See "Cross-validation scheme" in manuscript.**
	**21**	Level at which partitions are disjoint (e.g., image, study, patient, institution)	**No same patient data was allowed to be present in one train-test split across the cross-validation cods.**
Model	**22**	Detailed description of model, including inputs, outputs, all intermediate layers and connections	**See "Methods" in manuscript.**
	**23**	Software libraries, frameworks, and packages	**See "Methods" in manuscript and Supplemental.**
	**24**	Initialization of model parameters (e.g., randomization, transfer learning)	**As of utilized software library default parameters. See Supplemental for details.**
Training	**25**	Details of training approach, including data augmentation, hyperparameters, number of models trained	**Test tata was balanced to properly estimate balanced accuracy (BACC). For hyperparameters see Supplemental.**
	**26**	Method of selecting the final model	**No final model was selected. Study compared multiple classic ad quantum ML models to investigate their feasibilities and capabilities within the cohort.**
	**27**	Ensembling techniques, if applicable	**NA**
Evaluation	**28**	Metrics of model performance	**Confusion matrix analytics in test set.**
	**29**	Statistical measures of significance and uncertainty (e.g., confidence intervals)	**Confidence intervals (CI) of test predictive performances with 95% confidence levels. Pearson correlation with p=0.05 significance threshold.**

	**30**	Robustness or sensitivity analysis	**All confusion metrics including balanced accuracy were measured only in test subsets of the cross-validation scheme. CIs were compared in-between different ML methods.**
	**31**	Methods for explainability or interpretability (e.g., saliency maps), and how they were validated	**NA**
	**32**	Validation or testing on external data	**Cross-validation with train-test splits of single-center cohorts. Only test predictive performance values were reported.**
RESULTS			
DATA	**33**	Flow of participants or cases, using a diagram to indicate inclusion and exclusion	**NA**
	**34**	Demographic and clinical characteristics of cases in each partition	**NA**
Model Performance	**35**	Performance metrics for optimal model(s) on all data partitions	**Confusion matrix performance metrics in test subsets.**
	**36**	Estimates of diagnostic accuracy and their precision (such as 95% confidence intervals)	**Average test performance metrics across 10-folds. 95% CI for each performance metric.**
	**37**	Failure analysis of incorrectly classified cases	**NA**
DISCUSSION			
	**38**	Study limitations, including potential bias, statistical uncertainty, and generalizability	**Single-center cohorts. See "Discussion" in manuscript for details.**
	**39**	Implications for practice, including the intended use and/or clinical role	**Quantum ML is feasible to yield high-performing prediction models in both simulators and real hardware in collected cohorts.**
OTHER INFORMATION			
	**40**	Registration number and name of registry	**Clinical Trials: NCT02659527<br>See DOIs: 10.1007/s00259-020-05140-y, 10.2967/jnumed.117.202267, 10.2967/jnumed.117.202267 for details.**
	**41**	Where the full study protocol can be accessed	**Clinical Trials: NCT02659527<br>See DOIs: 10.1007/s00259-020-05140-y, 10.2967/jnumed.117.202267, 10.2967/jnumed.117.202267 for details.**
	**42**	Sources of funding and other support; role of funders	**Medical University of Vienna-funded project. L. Papp is the PI of an in-house grant "Quantum Image Analysis", funding 50k EUR for 1 year.**

[1] H.-Y. Huang, M. Broughton, M. Mohseni, R. Babbush, S. Boixo, H. Neven, and J. R. McClean, Nature Communications **12**, 2631 (2021).

[2] M. Abramowitz and I. A. Stegun, *Handbook of Mathematical Functions with Formulas, Graphs, and Mathematical Tables*, ninth dover printing, tenth gpo printing ed. (Dover, New York City, 1964).

[3] S. Gambs, "Quantum classification," (2008).

[4] D. Horn and A. Gottlieb, Phys. Rev. Lett. **88**, 018702 (2001).

[5] A. W. Harrow, A. Hassidim, and S. Lloyd, Phys. Rev. Lett. **103**, 150502 (2009).

[6] M. Schuld, A. Bocharov, K. M. Svore, and N. Wiebe, Phys. Rev. A **101**, 032308 (2020).

[7] M. Kieferová and N. Wiebe, Phys. Rev. A **96**, 062327 (2017).

[8] C. P. Williams, *Explorations in Quantum Computing*, 2nd ed. (Springer Publishing Company, Incorporated, 2008).

[9] R. R. Tucci, "A rudimentary quantum compiler(2cnd ed.)," (1999).

[10] M. Möttönen, J. J. Vartiainen, V. Bergholm, and M. M. Salomaa, Phys. Rev. Lett. **93**, 130502 (2004).

[11] S. Johri, S. Debnath, A. Mocherla, A. SINGK, A. Prakash, J. Kim, and I. Kerenidis, npj Quantum Information **7**, 122 (2021).

[12] S. Khatri, R. LaRose, A. Poremba, L. Cincio, A. T. Sornborger, and P. J. Coles, Quantum **3**, 140 (2019).
[13] K. Heya, Y. Suzuki, Y. Nakamura, and K. Fujii, "Variational quantum gate optimization," (2018).
[14] M. Schuld and F. Petruccione, *Supervised Learning with Quantum Computers*, 1st ed. (Springer Publishing Company, Incorporated, 2018).
[15] M. Cerezo, A. Arrasmith, R. Babbush, *et al.*, Nat Rev Phys **7**, 625–644 (2021).
[16] R. Wille, S. Hillmich, and L. Burgholzer, in *2020 IEEE International Symposium on Circuits and Systems (ISCAS)* (2020) pp. 1–5.
[17] J. A. Smolin and D. P. DiVincenzo, Phys. Rev. A **53**, 2855 (1996).
[18] N. Margolus, in *Collision-Based Computing*, edited by A. Adamatzky (Springer London, 2012).
[19] G. Song and A. Klappenecker, "The simplified toffoli gate implementation by margolus is optimal," (2003).
[20] H. Abraham *et al.*, "Qiskit: An open-source framework for quantum computing," (2021).
[21] S. Debnath, N. M. Linke, C. Figgatt, K. A. Landsman, K. Wright, and C. Monroe, Nature **536**, 63 (2016).
[22] S. Moradi, C. Brandner, C. P. Spielvogel, D. Krajnc, S. Hillmich, R. Wille, W. Drexler, and L. Papp, Scientific Reports **12** (2022), https://doi.org/10.1038/s41598-022-05971-9.
[23] C. M. Bishop and N. M. Nasrabadi, *Pattern recognition and machine learning*, Vol. 4 (Springer, 2006).
[24] M. Schuld, A. Bocharov, K. M. Svore, and N. Wiebe, Phys. Rev. A **101**, 032308 (2020).
[25] F. Pedregosa, G. Varoquaux, A. Gramfort, V. Michel, B. Thirion, O. Grisel, M. Blondel, P. Prettenhofer, R. Weiss, V. Dubourg, J. VanderPlas, A. Passos, D. Cournapeau, M. Brucher, M. Perrot, and E. Duchesnay, CoRR **abs/1201.0490** (2012), 1201.0490.
[26] V. Bergholm, J. A. Izaac, M. Schuld, C. Gogolin, and N. Killoran, CoRR **abs/1811.04968** (2018), 1811.04968.
[27] M. Q. Team, "Microsoft azure," ().
[28] S. Crain, C. Cahall, G. Vrijsen, E. E. Wollman, M. D. Shaw, V. B. Verma, S. W. Nam, and J. Kim, Communications Physics **2**, 1 (2019).
[29] I. Q. Team, "Ionq aria," ().
[30] M. Urbanek, B. Nachman, V. R. Pascuzzi, A. He, C. W. Bauer, and W. A. de Jong, Phys. Rev. Lett. **127**, 270502 (2021).
[31] A. Strikis, D. Qin, Y. Chen, S. C. Benjamin, and Y. Li, PRX Quantum **2**, 040330 (2021).
[32] K. C. J. Mongan J, Moy L, Radiol Artif Intell. **2** (2020), https://doi.org/10.1148/ryai.2020200029.
